# Predicting diet quality and food consumption at eating occasions using contextual factors: an application of machine learning models

**DOI:** 10.1186/s12966-025-01818-4

**Published:** 2025-11-04

**Authors:** Nancy R. Tran, Yuxin Zhang, Rebecca M. Leech, Sarah A. McNaughton

**Affiliations:** 1 Health and Well-Being Centre for Research Innovation, School of Human Movement and Nutrition Sciences, University of Queensland, St Lucia, QLD 4067 Australia; 2 School of Exercise and Nutrition Sciences, Deakin University, Geelong, VIC 3220 Australia; 3 Institute for Physical Activity and Nutrition (IPAN), School of Exercise and Nutrition Sciences, Faculty of Health, Deakin University, Melbourne Burwood Campus, 221 Burwood Highway, Burwood, VIC 3125 Australia

**Keywords:** Machine learning, Food intakes, Contextual factors, Eating occasion, Ecological momentary assessment, Young adults

## Abstract

**Background:**

Eating occasions (EOs) are important moments for dietary decision-making. Tailoring nutrition interventions to individuals’ behaviours and environmental contexts offers a promising way to modify eating habits at EOs and enhance diet quality. While machine learning (ML) is a useful tool for predicting behaviours, its potential in understanding food choices at EOs remains underexplored. This study uses ML to investigate whether contextual factors can predict food consumption at EOs and overall daily diet quality.

**Methods:**

Cross-sectional data from the Measuring Eating in Everyday Life Study (MEALS) were analysed. Participants (18-30y, *n* = 675) recorded intakes for 3–4 non-consecutive days using a Smartphone food diary app. EO-level contextual factors at each EO were recorded via the app while person-level contextual factors were collected via an online survey. Intakes were calculated as servings of vegetables, fruits, grains, meat, dairy, and discretionary foods, in accordance with the Australian Dietary Guideline. Diet quality was assessed via Dietary Guideline Index (DGI, 0-120). Gradient boost decision tree and random forest algorithms were used for hurdle prediction models, with lowest mean absolute error (MAE) as best performing. Mean absolute SHapley Additive exPlanations (SHAP) values were used to interpret the impact of each factor in explaining food consumption predictions.

**Results:**

Predictive models performed robustly, with MAE below half a serving for various food groups: 0.3 servings for vegetables, 0.75 for fruit, 0.28 for dairy, 0.55 for grains, 0.4 for meat, and 0.68 for discretionary foods per EO. This indicates small deviations between the model’s predictions and actual intakes. For overall daily diet quality, the model predictions deviated by 11.86 DGI points from the actual score. From mean absolute SHAP values, the importance of predictive factors varied across the six food groups, while for diet quality, the most influential factors were cooking confidence, self-efficacy, food availability, perceived time scarcity, and activity during consumption.

**Conclusion:**

ML provided valuable insights into predicting food consumption based on contextual factors. Future research should explore how ML can assist identify key factors that, when adjusted for individual behaviour patterns, encourage healthier eating habits.

**Supplementary Information:**

The online version contains supplementary material available at 10.1186/s12966-025-01818-4.

## Introduction

Poor diet quality is a preventable risk factor associated with non-communicable diseases, including obesity, hypertension and cardiovascular diseases, which together account for 74% of global death [1–4]. In Australia, young adults are particularly vulnerable, with only 2.1% of those aged 18–24 meeting the recommended intake of fruits and vegetables in 2022 [5]. Many eating habits that contribute to this inadequate diet quality are established during young adulthood, making this period crucial for intervention [6, 7]. Understanding the factors influencing dietary behaviours in this population is essential for developing effective nutritional strategies.

One approach to explore these behaviours is by examining eating occasions (EOs), which can capture both temporal and contextual aspects of food consumption. EOs include a wide range of eating events, from main meals to snacks and beverage-only occasions [8]. Temporal aspects refer to when eating occurs, such as time of day, frequency, and timing relative to other daily activities, which influence dietary choices and patterns [9, 10]. Alongside timing, the environment surrounding an EO, called contextual factors, often coincides with variations in what is consumed. Decisions about what to consume during EOs are shaped by contextual factors that operate at both the person level and the eating occasion level (EO-level). Person-level factors represent individual characteristics, such as age, gender, socioeconomic, and dietary preferences, while EO-level factors reflect the immediate context, such as location, social context, and activities at the time of eating [11–13]. Given that these decisions are often highly personalised and influenced by external circumstances [14], examining both person-level and EO-level contextual factors is key to understanding eating behaviours.

Given the complex data structure inherent in eating behaviours, machine learning (ML) offers new opportunities to explore eating patterns and develop small, achievable recommendations that may be more effective in promoting optimal diet quality. One approach to examining eating patterns is using Ecological Momentary Assessment (EMA) [15], which allows the collection of real-time data on eating behaviours and contextual factors through repeated self-reports using diaries or electronic devices. By reducing recall bias and capturing naturalistic, time-sensitive data, EMA capture the variability in food group consumption across EOs. For instance, certain foods like fruits may be consumed more frequently at snacks than at meals [9]. Understanding these fluctuations is needed for identifying how contextual factors influence food choices and intake at each EO. ML can analyse these detailed datasets to detect complex patterns and relationships, including non-linear associations and interactions among multiple variables, without the need to pre-specify them. For example, ML might reveal that individuals are more likely to consume vegetables when meals are planned and consumed in social settings, but less likely to do so when eating alone. By integrating person-level and EO-level contextual factors, ML models have the potential to enhance the precision of dietary interventions, offering recommendations tailored to individual needs and environmental contexts. Such personalised interventions, focused on the unique circumstances of each EO, could improve long-term dietary behaviours by making changes more relevant and actionable [16, 17].

The application of ML in diet-related research has shown significant promise in predicting eating behaviours and informing personalised nutrition strategies [18, 19]. However, existing studies have limitations, particularly in their ability to predict food consumption in free-living young adults. Some research has focused on single food groups, offering a limited view of overall dietary patterns and health outcomes [20, 21]. Others have used neural network models to predict diet adherence, but their complexity often limits interpretability for practical applications [22]. Additionally, while some studies have explored AI-based food substitutions, they have primarily assessed user acceptance and have yet to investigate improvements in diet quality [23]. Integrating contextual factors and all food group consumption at the level of EOs, alongside overall daily diet quality, may enhance the relevance and effectiveness of ML-based dietary recommendations.

This study aims to investigate the dietary behaviours of free-living young adults, focusing on food consumption at EOs using machine learning algorithms. Specifically, we apply various ML models to assess whether contextual factors during consumption can predict the quantities of food groups consumed at EOs among Australian young adults. Ultimately, this analysis aims to provide insights into the interaction between contextual factors, person-level and EO-level, and daily diet quality, contributing to the development of more personalised nutrition recommendations for this population.

## Methods

### Study design

This secondary analysis used data from the Measuring Eating in Everyday Life Study (MEALS), a cross-sectional study conducted between April 2015 and April 2016 to investigate eating patterns among young adults aged 18–30 years [24]. Detailed descriptions of the MEALS study design and dietary assessment methods are described elsewhere [7, 24]. Briefly, participants were recruited through both online (e.g., Facebook, Twitter) and offline (e.g., posters, flyers) methods. Inclusion criteria included owning a smartphone purchased in Australia, not being pregnant or lactating, speaking English as the primary household language, and residing in Victoria, Australia.

Participants provided informed written consent and were asked to complete an online questionnaire via Qualtrics, followed by four non-consecutive days of food intake recording using the “FoodNow” smartphone application [25]. Upon study completion, they received a $25 AUD voucher. All participants provided informed written consent to participate, and ethical approval was granted by the Deakin University Human Ethics Advisory Group, Faculty of Health (HEAG-H 11_2015). The STrengthening the Reporting of OBservational studies in Epidemiology – Nutritional Epidemiology statement [26] was included with this manuscript (Additional file 5).

Participants who reported fewer than three non-consecutive days of dietary assessment were excluded from the analysis, resulting in a final sample of 675 young adults.

### Dietary assessment

Using the FoodNow food dairy app (reported in detail in Pendergast et al. [25]), participants reported their food intake in near-real time over four non-consecutive days, including at least one weekend day within a two-week period. For each EO, they provided images along with text descriptions of the types and quantities of all foods and beverages consumed with quantities reported in household measures. To minimise under-reporting, the app included prompts for users to report any uneaten foods or drinks.

Participants received reminders to log any missed foods or meals if no EO had been recorded within three hours during waking hours, as well as a daily prompt at the end of each day. The app has been validated in previous research, demonstrating strong agreement with objectively measured energy expenditure (intra-class correlation = 0.75) [25]. Trained nutritionists coded the dietary entries, including food images, text, and voice descriptions, and matched them to items in the Australian Food, Supplements, and Nutrient Database 2011–2013 (AUSNUT13) [27]. A duplicate review process was carried out to ensure the accuracy of the data [25].

In addition to reporting their food and beverage intake, participants also recorded the starting time and type of each EO (e.g., breakfast, lunch, dinner, snack) through the app. They also recorded the eating location, who was present, what they were doing, and where the foods were sourced from. For this analysis, any food or drink consumed simultaneously and contributing a total minimum energy of 210 kJ was considered one EO.

### Food groups classification and servings calculation

Each reported food or beverage was first classified as discretionary or non-discretionary, using the Australian Bureau of Statistics Discretionary Food List [28]. A serving of discretionary foods was defined as 600 kJ energy intake with fibre. Non-discretionary foods were grouped into five categories: vegetables, fruits, grains, meats and alternatives, and dairy and alternatives based on the Australian Dietary Guidelines (ADG). Serving sizes were estimated using the ADG Food Group database [29]. The final outcomes used for prediction were log-transformed servings per EO for each food group, calculated by multiplying the actual value by the natural log plus one.

### Diet quality

Diet quality was measured using the Dietary Guideline Index (DGI), which assesses adherence to the 2013 ADG [30–32]. The DGI has been adapted for application in 24-hour recall data and comprises of 12 items (Additional file 1). Each item is scored based on adherence to food-based recommendations, with a maximum score of 10 points per item. Total DGI scores range from 0 to 120, with higher scores indicating better diet quality [31]. Daily DGI scores were calculated for each participant.

### Contextual factors

Contextual factors were categorised into person-level and EO-level, based on existing literature on their potential influence on food consumption [13]. A detailed description of these factors is provided in Additional file 2.

Person-level contextual factors were grouped into intrapersonal, socio-environmental, and physical-environmental categories. These factors were collected via the online questionnaire, using existing and previously validated questions. Intrapersonal factors included variables such as sex/gender (female, male), age [18–30], country of birth (Australia, other), gross average income (AUD) per week (< 120, $120 - $499, $500 - $999, >= $1000), level of education (low – high school or less, medium – TAFE/vocational qualification, high – university qualifications), smoking status (never smoked, past smoker, current smoker), meeting Australia’s Physical Activity and Sedentary Behaviour Guidelines (yes − 150 min of moderate-intensity physical activity or 75 min of vigorous physical activity per week, no – less than 150 min of moderate-intensity physical activity or 75 min of vigorous physical activity per week) [33]. The level of physical activity was measured by the International Physical Activity Questionnaire (IPAQ) [34].

Other person-level contextual factors relating to intrapersonal category included food meal preparation behaviour (score from 12 to 84, with higher scores indicating more frequent engagement in food preparation tasks such as planning and cooking) [35], food shopping behaviour (score from 0 to 21, with higher scores indicating greater involvement in planning and performing food shopping activities) [36, 37], self-efficacy (score from 17 to 85, with higher scores indicating stronger confidence in one’s ability to eat healthily across various situations) [38], and cooking confidence (score from 0 to 21, with a higher score indicating greater cooking confidence) [39]. Perceived time scarcity (score from 4 to 16, with higher scores indicating a greater sense of time pressure)) and food choice barriers (score from 0 to 11, with higher scores indicating more perceived obstacles to making healthy food choices) were assessed using questions from the Project EAT study [35]. Food shopping behaviour was measured by asking participant if they shopped for food or groceries [36] and whether they wrote a shopping list before shopping [37]. Adapted versions of Sallis et al. self-efficacy scale, and the United Kingdom’s 1993 Health and Lifestyle Survey were used to measure self-efficacy, and cooking confidence, respectively [38–40].

Person-level social-environmental factors included family and friend social support (composite score from 3 to 15, with higher scores indicating greater perceived support for healthy eating from family and friends) [39, 41]. Physical-environmental factors measured proximity and access to food destinations (composite score from 16 to 63, measured by Neighbourhood Environment Walkability Scale, with higher scores indicate closer proximity and greater access) [42], food availability (composite score, from 0 to 12, higher score indicate greater food availability) [39], living situation (living alone, living with family, living with friends/flatmates) and area-level socioeconomic status (Socio-economic Index for Areas (SEIFA): low [least disadvantage], medium, high [most disadvantage]) [43].

EO-level factors, captured in real-time using the FoodNow app, were classified as social and environmental, based on previous measures [13]. EO-level social factors included presence of others (alone, with friends, with other people) and activity at EO (nothing, visiting family/friends, screen-based activity, other). EO-level environmental factors that measure the surrounding food environment at the EO included location of consumption (home, outside of the home, in transit, other) and location of food purchase (supermarkets, convenience stores, restaurants, other). EO-level preparation factors describe factors relating to the preparation of the foods consumed at an EO; for each food recorded in the app, participants reported whether it was homemade or not (yes, no).

### Statistical analysis

**Fig. 1:**
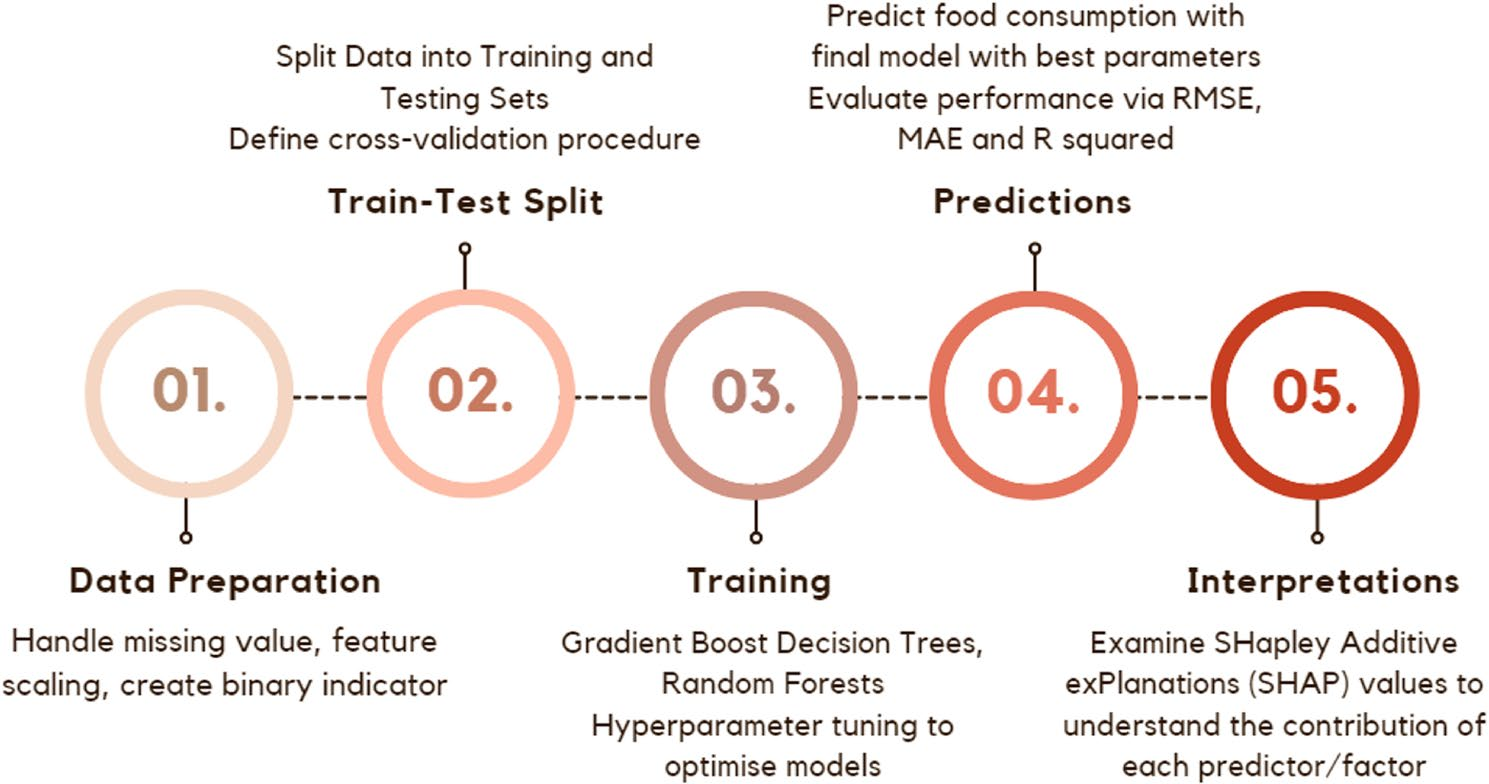
Flowchart of machine learning stages

Absolute frequencies (%) and means (95% CI) were used to summarise person-level contextual factors. Welch two sample t-test, and Pearson’s chi-squared test with Yates’ continuity correction were used to compare the characteristics of participants who provided dietary data for three days versus those who provided data for four days. ML techniques were used to predict food group consumption at EOs and overall daily diet quality (Fig. 1). Stochastic Gradient Boosting Decision Tree (SGBDT) and Random Forest (RF) models were chosen for their ability to handle the complexity of the data (i.e. zero-inflated and skewed data at EO) [44–49]. Four predictive models were applied: SGBDT, RF, and their hurdle model extensions to address zero-inflated data, as summarised in Table 1. A hurdle model is a two-part approach that first predicts the likelihood of any food consumption (non-zero vs. zero intake) and then estimates the amount consumed when intake occurs, making it suitable for data with a high frequency of zeros.

**Table 1:** Model and algorithm used for predicting food group consumption at eating occasions and daily diet quality

Model	Algorithms description
Stochastic Gradient Boosting Decision Tree (SGBDT)	An ensemble learning algorithm that builds a sequence of decision trees, where each tree is trained to correct the errors of the previous one. SGBDT focuses on optimising predictive accuracy by minimising a loss function, making it highly suitable for complex, non-linear relationships. It tends to outperform other algorithms when there is a need to capture fine-grained patterns in the data. However, SGBDT can be more prone to overfitting, especially in noisy data, unless properly regularised. This method was selected due to its ability to handle intricate dependencies between contextual factors and dietary outcomes, and its proven effectiveness in health-related prediction tasks.
Random Forest (RF)	RF constructs multiple independent decision trees on bootstrapped samples of the data and averages their predictions, making it more robust to overfitting compared to SGBDT. RF maintaining high accuracy when the data contains noisy or semi-continuous variables, as the aggregation of trees reduces variance. It is computationally more efficient in some cases and less sensitive to hyperparameter tuning. RF was chosen for its reliability in handling the complex, varied nature of dietary data and its ability to generalise well across different contexts, even when the underlying relationships between variables are less clear-cut.
Hurdle SGBDT	A two-part model consisting of a binary SGBDT to predict the likelihood of any food intake (non-zero vs. zero consumption) and a continuous SGBDT to predict the amount of food consumed, conditional on the binary outcome being non-zero. The first part of the model deals with whether consumption occurred, and the second part estimates how much was consumed when consumption did occur. This method is suited for handling zero-inflated data where non-consumption is common.
Hurdle RF	Using the same framework as the Hurdle SGBDT model but uses RF algorithms for both the binary and continuous parts of the model. It leverages the strengths of RF in managing data with high variability and multiple predictors.

ML techniques presented in Fig. 1 began with data preparation, imputing missing values in the predictor variables (Additional file 3) and transforming food group consumption (natural log of servings) while daily DGI scores remained in its original scale. To facilitate robust model training and evaluation, a custom function was developed to create repeated folds. This function utilised participant IDs to ensure that data from the same individual were not included in both training and testing sets, thereby preventing data leakage. A hyperparameter grid was defined to optimise the parameters for the model. Custom repeated folds were then generated for cross-validation, with a control object specifying repeated 10-fold cross-validation and enabling hyperparameter tuning through grid search. Therefore, total training runs for each SGBDT and RF model were 6480 times and 300 times, respectively. Parallel computing backend was used to enhance processing efficiency by utilising multiple cores.

After training models, predictions were generated and transformed back to the original scale using the exponential transformation. Performance metrics—including Root Mean Squared Error (RMSE), Mean Absolute Error (MAE), and R-squared—were computed to evaluate model accuracy on the original scale of the outcomes. RMSE measured the average magnitude of error in the model’s predictions, with lower values indicating closer alignment with actual values. MAE measured the average absolute difference between predicted and actual values, with lower values indicating better performance. R squared assesses the proportion of variance in the outcome explained by the predictors, ranging from 0 (no explanatory power) to 1 (complete explanatory power). Both RMSE and MAE values were transformed back to original scale (servings). Models with lowest MAE were considered as best performing model as intake at EO contains outliers that disproportionately influence the overall distribution.

Following performance evaluation, model interpretation was conducted using SHapley Additive exPlanations (SHAP) values. SHAP values provided insights into how the presence or absence of each feature impacted predictions [50]. This assessed the contribution of each feature/factor to the model’s output at both local (individual EOs) and global (all EOs) levels. Mean absolute SHAP values were computed for different levels of data aggregation, including individual EO, daily EOs, and all EOs across participants. Higher mean absolute SHAP values indicate greater influence of a predictor on model predictions, enabling an understanding of which contextual factors most strongly shaped food intake predictions across levels. This approach enabled a thorough examination of feature importance, enhancing model interpretability. All analyses were conducted in R 4.3.3 and RStudio, using various packages (dplyr, mice, caret, iml, doParallel, ggplot2, ggbump) [51].

## Results

This analysis examined a sample of 675 young adults, with the majority (*n* = 582) reporting dietary intake over four non-consecutive days, while a smaller subset (*n* = 93) reported intake over three days (Table 2). The average age of participants was similar across dietary reporting groups (3 vs. 4 days), with a mean of 24.24 years overall. The sample was predominantly females (73.33%) and born in Australia (75.11%). The weekly income distribution showed variation between dietary reporting groups, with those reporting four days of intake more likely to fall into lower (<$120) and middle ($120-$499) income brackets (*p* = 0.01). Education levels were comparable across dietary reporting groups, with approximately 59% having completed a tertiary degree. Intrapersonal and social-environmental characteristics also showed few differences between the dietary reporting groups. Physical activity levels were similar, with about 63% of participants meeting physical activity guidelines, and smoking rates were low across both groups, with fewer than 20% reporting current or past smoking. Scores for meal preparation, food shopping, and self-efficacy were consistent between three- and four-day reporters. Additionally, social support from family and friends was comparable, as were participants’ living arrangements.

**Table 2:** Comparison of participants characteristics (person-level contextual factors) by number of consumption days reported (Three days vs. Four days)

	Total sample (*n* = 675)	Three days reported (*n* = 93)	Four days reported (*n* = 582)	*p*-value^1^
Intrapersonal characteristics				
Age, mean (95%CI)	24.24 (24.0, 24.5)	24.37 (23.63, 25.11)	24.23 (23.94, 24.51)	0.7244
Sex, n (%)				
Male	180 (26.67)	29 (31.18)	151 (25.95)	0.3501
Female	495 (73.33)	64 (68.82)	431 (74.05)	
Country of birth				
Australia	507 (75.11)	64 (68.82)	443 (76.12)	0.1668
Other	168 (24.89)	29 (31.18)	139 (23.88)	
Gross average income (AUD) per week				
<$120/w	134 (21.24)	18 (19.35)	116 (19.93)	0.0137
$120-$499/w	229 (36.29)	19 (20.43)	210 (36.08)	
$500-$999/w	128 (20.29)	22 (23.66)	106 (18.21)	
>=$1000/w	140 (22.19)	23 (24.73)	117 (20.10)	
Level of education				
Low - Year 12 or less	192 (28.44)	28 (30.11)	76 (13.06)	0.802
Mid - Dip./Cert. or Trade/Apprenticeship	86 (12.74)	10 (10.75)	164 (28.18)	
High - Tertiary degree	397 (58.81)	55 (59.14)	342 (58.76)	
Smoking status				
Never	543 (80.44)	75 (80.65)	468 (80.41)	0.1755
Past	71 (10.52)	6 (6.45)	65 (11.17)	
Current	61 (9.04)	12 (12.90)	49 (8.42)	
Physical Activity^1^				
Met	427 (63.26)	61 (65.59)	366 (62.89)	0.6991
Not met	248 (36.74)	32 (34.41)	216 (37.11)	
Meal Preparation behaviour score, mean (95% CI)	63.22 (62.49, 63.95)	63.01 (61.03, 64.99)	63.25 (62.47, 64.04)	0.8242
Food shopping behaviour score, mean (95% CI)	31.32 (30.77, 31.86)	30.42 (29.05, 31.78)	31.46 (30.86, 32.05)	0.1737
Self-efficacy score, mean (95% CI)	54.41 (53.39, 55.43)	54.86 (52.06, 57.66)	54.34 (53.25, 55.43)	0.7349
Cooking confidence score, mean (95% CI)	15.34 (15.04, 15.65)	14.80 (14.01, 15.58)	15.43 (15.10, 15.76)	0.1452
Perceived time scarcity, mean (95% CI)	7.11 (6.91, 7.30)	7.33 (6.81, 7.86)	7.07 (6.86, 7.28)	0.3597
Food choice barriers, mean (95% CI)	2.07 (1.96, 2.19)	2.15 (1.84, 2.46)	2.06 (1.94, 2.18)	0.5984
Social-environmental factors				
Social support from family, mean (95CI %)	9.49 (9.28, 9.69)	9.70 (9.15, 10.25)	9.45 (9.23, 9.68)	0.4153
Social support from friends, mean (95CI %)	6.91 (6.71, 7.12)	6.58 (6.05, 7.12)	6.97 (6.75, 7.19)	0.1882
Physical-environmental factors				
Living situation				
Parents/family	246 (36.44)	34 (36.56)	212 (36.43)	0.8694
By myself	76 (11.26)	11 (11.83)	65 (11.17)	
Partner/spouse	174 (25.78)	21 (22.58)	153 (26.29)	
Other sharing arrangement	179 (26.52)	27 (29.03)	152 (26.12)	
Food nearness score, mean (95% CI)	44.36 (43.58, 45.14)	45.20 (43.09, 47.30)	44.23 (43.39, 45.06)	0.4055
Food availability score, mean (95% CI)	12.60 (12.41, 12.79)	12.31 (11.69, 12.92)	12.65 (12.45, 12.84)	0.3126
SEIFA^2^				
Low	106 (13.87)	12 (12.90)	78 (13.40)	0.3628
Medium	193 (25.26)	20 (21.51)	153 (26.29)	
High	461 (60.34)	60 (64.52)	350 (60.14)	
Eating occasions, n	11,497	1018	10,479	
EOs per person, mean (95% CI)	17.03 (16.52, 17.55)	10.83 (9.96, 11.70)	18.04 (17.49, 18.58)	< 0.001

^1^*p*-value – Welch two sample t-test, and Pearson’s chi-squared test with Yates’ continuity correction were used to compare the characteristics of participants who provided dietary data for three days versus those who provided data for four days

^2^SEIFA – Socio-Economic Indexes for Areas

### Model prediction performance

Table 3 presents the performance metrics of all models used to predict the consumption of six food groups and overall diet quality. The best-performing models, based on mean absolute error (MAE) scores, were the RF and Hurdle RF models. The RF models achieved the lowest MAE for predicting vegetable, fruit, dairy and alternatives and discretionary food intake at EOs (MAE: 0.30, 0.75, 0.28 and 0.59 servings, respectively). Meanwhile, the Hurdle RF models had the lowest MAE for predicting grains, meat and alternatives at EOs (MAE: 0.55, and 0.40 servings, respectively). The Hurdle RF model also exhibited the highest R-squared values across all prediction models.

It is important to note that hurdle models were not applied to daily diet quality predictions, as this outcome is a continuous score measured at the daily level and contains no zero values, unlike food consumption at EOs, which frequently includes zero values. The hurdle models estimated both the probability of intake at EOs, and the amount consumed at those EOs. Therefore, the RF and SGBDT models alone were used for predicting daily diet quality, where RF demonstrated slightly better performance metrics compared to the SGBDT model.

**Table 3:** Performance metrics of all models used to predict food group consumption and daily diet quality. This table presents the performance of four machine learning models—Stochastic gradient boosted decision trees (SGBDT), random forests (RF), hurdle SGBDT, and hurdle RF—applied to predict intake (serves) of individual food groups and daily diet quality scores. Outcomes include fruit, vegetable, grain, meat and alternatives, dairy and alternatives, discretionary foods, and an overall daily diet quality measure. For each outcome, the model with the lowest MAE (best performance) is marked with an asterisk (*) with other metrics bolded. Lower RMSE and MAE, and higher R² values indicate better model performance.

		Performance metrics		
Outcome	**Model**	RMSE^1^	MAE^2^	R squared
Fruits	SGBDT	0.63	0.35	0.049
	RF	**0.56**	0.30*	0.418
	Hurdle SGBDT	0.71	0.59	0.023
	Hurdle RF	0.69	0.44	**0.598**
Vegetables	SGBDT	1.57	0.94	0.099
	RF	1.33	0.75*	0.463
	Hurdle SGBDT	**1.12**	0.99	0.031
	Hurdle RF	1.54	1.00	**0.517**
Grains	SGBDT	1.29	0.96	0.096
	RF	1.03	0.72	0.496
	Hurdle SGBDT	0.94	0.85	0.021
	Hurdle RF	**0.82**	0.55*	**0.616**
Meat and alternatives	SGBDT	0.80	0.52	0.097
	RF	0.72	0.45	0.439
	Hurdle SGBDT	0.69	0.59	0.022
	Hurdle RF	**0.62**	0.40*	**0.632**
Dairy and alternatives	SGBDT	0.49	0.33	0.055
	RF	**0.43**	0.28*	0.441
	Hurdle SGBDT	0.57	0.50	0.017
	Hurdle RF	0.44	0.30	**0.562**
Discretionary foods	SGBDT	1.38	0.79	0.129
	RF	1.12	0.59*	0.552
	Hurdle SGBDT	**0.83**	0.68	0.027
	Hurdle RF	1.23	0.72	**0.620**
Daily diet quality	SGBDT	14.57	11.96	0.113
	RF	**14.41**	**11.86**	**0.119**

*Best performing. Bold – best performing metrics

^1^RMSE - Root Mean Squared Error

^2^MAE - Mean Absolute Error

### Global (all EOs) feature importance of best performing models

Table 4 reports the top five global (or overall) feature importance rankings for the best-performing models, as determined by mean absolute SHAP values. These values provide a comprehensive measure of factors contribution to the models’ predictions. Scores are reflective of its influence in predicting food group consumption at EOs across all observed individuals. Global feature importance offers an overarching perspective on the model’s behaviour by summarising the average impact of each factor across the entire dataset. This means that factors with high global SHAP values consistently influence predictions across many EOs and participants. In contrast, local feature importance refers to the contribution of factors to the model’s output for a specific instance, such as a particular EO or a day for an individual participant. Interpreting local SHAP values helps identify which factors most strongly influenced the prediction in that unique context, capturing individual variations and transient influences. Thus, while global importance identifies the key drivers on average, local importance provides insight into personalised and context-specific influences on dietary behaviour.

For predicting fruit intake at EOs, the top five features are primarily related to the person (person-level contextual factors), including two intrapersonal factors (cooking confidence and food choice barriers), one socio-environmental factor (social support from family), one physical environmental factor (food availability), and one factor relating to personal belief (self-efficacy). These factors were all equally important in the model for predicting fruit consumption (mean absolute SHAP = 0.0005). For predicting vegetable intake, the most influential factors were SEIFA and income (0.0035 and 0.0035), which are physical environmental and socio-economic factors relating to the person. For predicting dairy consumption, the factor with the highest mean absolute SHAP value was education (0.0012).

For grains, meats and their alternatives, and discretionary food groups, the feature importance in predicting consumption are divided into two parts (Table 4). The first part describes the factors’ importance in predicting the occurrence of consumption for these food groups. They are activity during consumption (an EO-level factor), cooking confidence (a person-level factor), and place of purchase (an EO-level factor) for grains, meats, and discretionary foods (0.003, 0.001, and 0.0024), respectively. Once consumption occurred, the factors with the highest scores for predicting the amount consumed were food choice barriers and living situation for grains (both 0.0008); smoking status and place of purchase for meats and their alternatives (both 0.0014); and place of consumption and perceived time scarcity for discretionary foods (both 0.0037).

For daily diet quality score, the top five features are primarily related to the person, with one factor relating to the EO (cooking confidence, self-efficacy, food availability, perceived time scarcity, activity at consumption: 0.0814, 0.08, 0.772, 0.076, and 0.0753, respectively).

**Table 4:** Top five factors for best performing models to predict food intakes at eating occasions and daily diet quality, using mean absolute SHAP values

Outcome	Occurrence		Amount	
	Feature	Mean SHAP	Feature	Mean SHAP
Fruits*	-	-	Self-efficacy	0.0005
	-	-	Food availability	0.0005
	-	**-**	Cooking confidence	0.0005
	-	-	Social support from family	0.0005
	-	-	Food choice barriers	0.0005
Vegetables*	-	-	SEIFA	0.0035
	-	-	Income	0.0035
	-	**-**	Social support from family	0.0034
	-	-	Proximity and access to food destinations	0.0034
	-	-	Age	0.0033
Grains	Activity at consumption	0.0030	Food choice barriers	0.0008
	Education	0.0024	Living situation	0.0008
	Country of birth	0.0023	Age	0.0007
	Food availability	0.0023	Social support from friends/colleagues	0.0007
	Place of purchase	0.0022	Perceived time scarcity	0.0007
Meat and alternatives	Cooking confidence	0.0010	Smoking status	0.0014
	Food choice barriers	0.0009	Place of purchase	0.0014
	Proximity and access to food destinations	0.0009	Food availability	0.0013
	education	0.0008	Food choice barriers	0.0013
	Country of birth	0.0008	Income	0.0013
Dairy and alternatives*	-	-	Education	0.0012
	-	-	PA	0.0011
	-	**-**	Country of birth	0.0011
	-	-	Food choice barriers	0.0011
	-	-	Food availability	0.0011
Discretionary foods	Place of purchase	0.0024	Place of consumption	0.0037
	Social support from friends/colleagues	0.0023	Perceived time scarcity	0.0037
	Education	0.0022	Social support from family	0.0036
	Proximity and access to food destinations	0.0021	Age	0.0036
	Country of birth	0.0019	Proximity and access to food destinations	0.0036
Daily diet quality*	-	-	Cooking confidence	0.0814
	-	-	Self-efficacy	0.0800
	-	**-**	Food availability	0.0772
	-	-	Perceived time scarcity	0.0760
	-	-	Activity at consumption	0.0753

*The best performing for these outcomes are random forest model. They do not have two parts (occurrence and amount) like Hurdle models

### Local (individual EOs) feature importances of best-performing models

Understanding the local (individual EOs) feature importances of the best-performing models may help tailor dietary interventions to people’s real-life behaviours. By examining mean absolute SHAP values, which indicate how much each factor contributes to the model’s predictions, we can gain insights at multiple levels: a single EO, a full day, or across multiple days for the same individual. At the level of a single EO, SHAP values highlight immediate drivers of food choices, such as social and environmental factors. When considered across a full day, they show how different situations combine to shape overall eating patterns. When analysed over several days, they identify broader behavioural trends and personal influences over time, supporting the development of personalised interventions. As an example, we examined how the importance of different factors changed in predicting fruit and vegetable consumption. This analysis showed that the influences on eating these foods vary depending on whether we consider one EO, a whole day, or behaviours across time. Results for other food groups and overall diet quality are presented in Additional File 4.

**Fig. 2:**
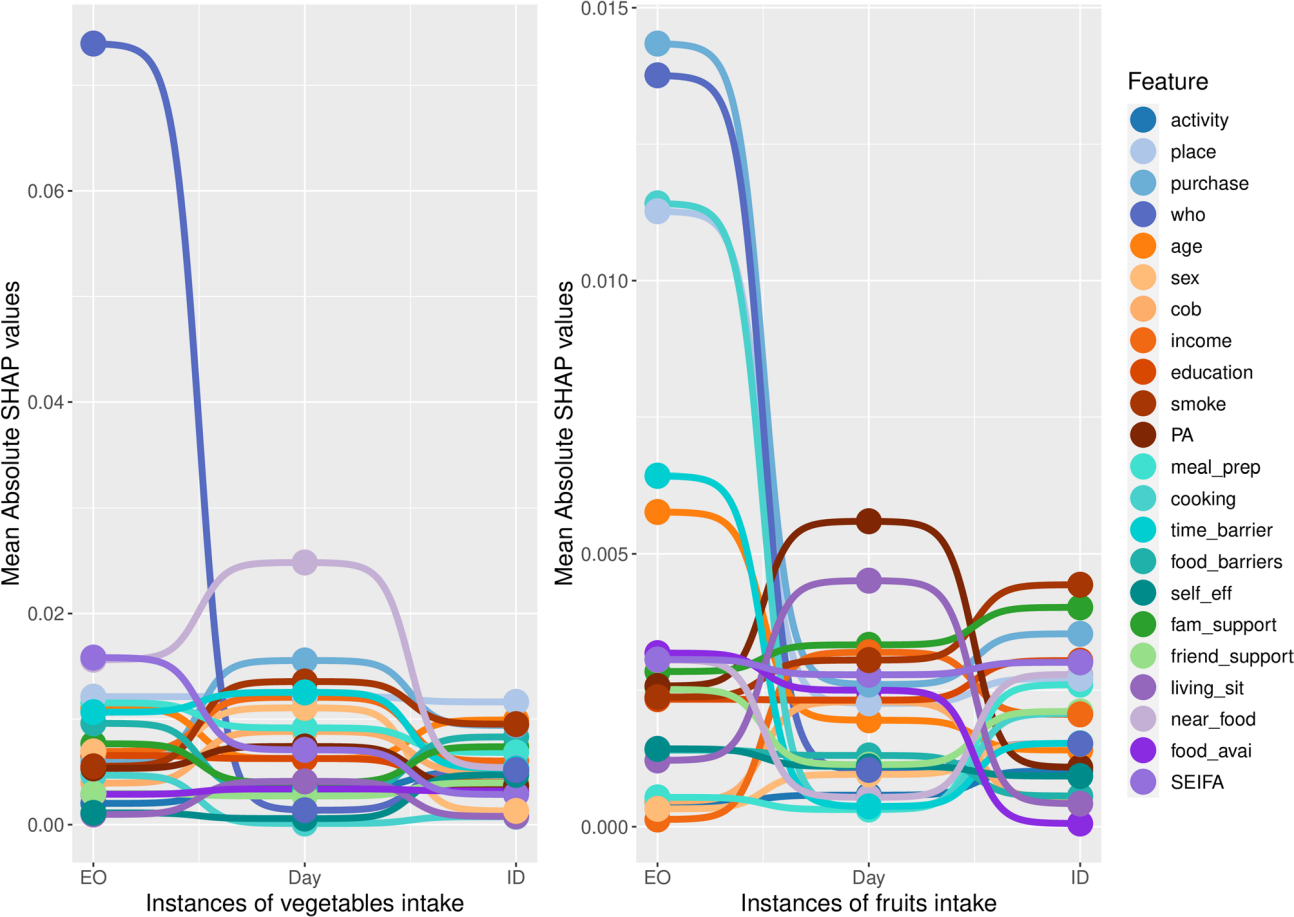
Feature importance based on mean absolute SHAP values for best predicting models (Random Forest) for vegetables & fruit intake. Results presented were randomly chosen to show changes in importance of features/predictors at different level of instance; EO has one eating occasion, Day included all eating occasion in one day, and ID included all eating occasions recorded by one individual (ID = 161). The mean absolute value of each feature over all instances (all eating occasions recorded from all individuals) is not presented but reported in Additional file 3. A feature/predictor with a higher mean absolute SHAP values contributes more significantly to the prediction on average. Eating occasion level factors such as activity (activity at consumption), place (place of consumption), purchase (place of purchase), and who (presence of others) have higher importance at predicting vegetables and fruits intakes in one eating occasion, and lower in importance when predicting intakes at eating occasion over a whole day, further lowering over several days. Eating occasion–level factors (e.g., activity, place, purchase source, social context) are shown in shades of blue. Person-level intrapersonal contextual factors are represented in orange and turquoise hues. Person-level socio-environmental factors are shown in green. Person-level physical environmental factors are shown in purple

To illustrate how contextual factors differ across levels of analysis, we examined the top contributing features for fruit and vegetable intake using SHAP values for participant ID 161. For fruit intake, the top five features (or factors) for a randomly selected EO reported by participant ID 161 were place of purchase, person present at consumption, cooking confidence, place of consumption, and perceived time scarcity (Fig. 2). When aggregating across all EOs reported by this participant in one day, the top factors were physical activity, living situation, social support from family, income level, and smoking status. Over 4 non-consecutive day period, the top five factors were smoking status, social support from family, place of purchase, education level, and SEIFA (Additional File 4, Table A4.1). While, for vegetable intake, the top factors for one EO included person present, SEIFA, food proximity and access, place of consumption, and meal preparation behaviour. Across one day of EOs, the top five factors were proximity and access to food, place of purchase, smoking status, perceived time barriers, and place of consumption. When examining all EOs over multiple days for participant ID 161, the top five factors were place of consumption, age, smoking status, place of purchase, and food choice barriers (Additional file 4, Table A4.2).

## Discussion

This study investigated the predictive ability of ML models to predict food group consumption at EOs and daily diet quality by learning from contextual factors. Our results highlighted the potential of ML to address challenges in dietary recommendation in personalised nutrition. ML models could predict food group consumption with low prediction errors, showcasing their ability to capture variability in dietary behaviours. Additionally, hurdle ML modelling was explored as a complementary approach to examine contextual factors influencing both the occurrence and quantity of food consumption, offering nuanced insights into eating behaviours. By comparing between traditional ML and Hurdle ML, this study enhances the potential for developing precise and adaptable nutrition recommendations that respond to immediate contexts while supporting broader dietary goals.

In this study, ML models demonstrated strong predictive performance across various food groups, with mean prediction errors consistently below half a serving. This highlights their potential as a robust methodological approach for addressing challenges in personalised nutrition, particularly in tailoring effective dietary recommendations. Challenges of dietary recommendation includes tailoring effective recommendation to individual due to the inherent variability and enhance the likelihood of sustained behavioural change [52]. This variability can occur within the same individual, both between EOs within a single day and across different days, reflecting the dynamic nature of dietary behaviours. By predicting food group consumption and addressing the inherent variability in dietary behaviours (ranging from no intake to six servings at a single EO), ML models have the potential to capture unique behavioural patterns and adapt to outliers. This adaptability allows for tailored recommendations that address both immediate contexts (e.g., specific meal choices) and long-term dietary goals, enhancing the likelihood of sustained behavioural change.

The findings of factor importance (SHAP scores) also align with existing literature on key contextual factors that influence food consumption and diet quality. For example, physical-environmental factors like food availability, particularly for fruits and discretionary foods, have been associated with consumption patterns [53]. Similarly, location of consumption was significant for discretionary food intake, supporting studies that link unhealthy eating behaviour to specific environments. For instance, one study found that eating while working on a computer or in an office setting was associated with higher consumption of sandwiches and sweets [54], while another classified locations like home, work, or away-from-home into groups, showing that non-designated eating places (e.g., in transit) led to unhealthy snacking [55]. Research also shows that consumption of meat and sugar-sweetened beverages is more likely in restaurants or cafés than at home [7, 56]. However, SHAP score allowed for a more nuanced understanding by revealing how these factors interact at different levels of analysis. For example, while location of consumption influenced discretionary food intake at individual EOs, its impact on daily diet quality was diminished by broader factors such as cooking confidence and self-efficacy. This finding may explain the discrepancies in the literature by providing insights into both immediate and cumulative influences on dietary behaviour, emphasising the complexity of eating patterns.

Our study demonstrates several key distinctions from prior research relating to ML applications in nutrition, by focusing on contextual factors and their impact at multiple levels of analysis. From a recent systematic review [57], studies predominantly aimed to personalise dietary plans for managing chronic diseases [58–61], or relied on household data or food images to infer nutritional recommendations. Unlike prior research, our study explores both at person- and EO- level and focused on the behavioural dimensions of dietary intake rather than the nutritional requirements. The inclusion of both the likelihood of consumption and the quantity consumed in the models highlights the potential of ML to facilitate behaviour change, moving beyond broader dietary goals or disease-specific nutrition plans. Global and local feature importance analyses using SHAP scores allowed for a nuanced understanding of how contextual factors influence eating patterns across varying levels of granularity, such as individual EOs, daily patterns, and individuals’ behaviours, an aspect underexplored in previous research.

Hurdle modelling was employed to deepen the understanding of dietary behaviours by separately examining the occurrence and quantity of food consumption, providing nuanced insights into each aspect of eating behaviour. While hurdle models have been used to predict energy intake [62], this study extends their application to servings of specific food groups. Our Hurdle RF model achieved higher R-squared values compared to other models, indicating its ability to explain a greater proportion of variability in food group consumption. However, for fruits, vegetables, and dairy, the RF model outperformed hurdle models in terms of MAE and RMSE, likely because it integrates the entire decision-making process into a unified framework, capturing the full influence of contextual factors.

By tailoring interventions to address both the likelihood of consumption and the quantity consumed, it becomes possible to precisely target the specific behaviours that an individual (or user) wishes to change. If an individual aims to reduce discretionary food consumption, the first part of the model can identify key influences, such as meal preparation behaviours, enabling personalised strategies like meal planning. Conversely, for reducing portion sizes, the second part of the model can target factors like social settings or dining out, allowing for actionable advice such as mobile prompts to limit overconsumption. This type of hurdle modelling approach offers a unique and highly personalised method for improving eating behaviours. This level of customisation in dietary recommendation for individuals have found to increase the probability of accepting the suggestions [23].

Understanding shifts in factor importance across different analytical levels enhances both the precision and credibility of personalised nutrition systems. At the level of a single EO, factors such as place of purchase and social presence highlight immediate influences on food choices. These insights reflect the situational influences that drive momentary decisions [7, 13]. When aggregated over a day, broader patterns emerge that reveal how contextual factors, such as physical activity (for fruit and discretionary foods) and proximity and access to food (for vegetables), influence eating behaviours throughout the day. These insights enable the development of recommendations tailored to recurring challenges or habits that manifest over a 24-hour period. At the individual level, overarching factors like socio-economic status or self-efficacy provide a deeper understanding of long-term behavioural trends. This multi-level analysis builds trust among experts by improving the interpretability of model outputs [63]. Understanding the reasoning behind model output allows recognition of model bias but also introduces the ability to embed behaviour change theories and inclusion of experts in the design of the dietary recommendations.

### Strengths and limitations

The major strengths of this study include the comprehensive assessment of intakes from the five food groups and discretionary foods at EOs, alongside contextual factors, in a relatively large sample of young adults. Findings of the present study represent a fundamental initial phase towards the development of customised recommendations, aimed at understanding and influencing the dietary behaviour of young adults. The study also employed novel, near-real-time measures of food intake with the amounts of food consumption calculated to align with the Australian Dietary Guidelines, ensuring the findings are relevant for experts and policies. Furthermore, the application of ML models provided a novel methodological contribution to the field of eating behaviours research.

However, there are important limitations to consider. The sample population, predominantly composed of females with high education and income levels, may not represent the broader population of Australian young adults, limiting the generalisability of the findings. In predicting food group consumption, reducing dimension (i.e. contextual factors) is important to improve model performance and efficiency. Our ML models reflect what was known in previous studies, using the same population. The ability of these ML model to be transferable to other populations maybe limited when complex interactions between contextual factors and individual characteristics are not explicitly identifiable in advance. Another limitation of this study is the moderate predictive ability for the adjusted DGI-2013 total score (range: 0–120), with a MAE of 11.86 points. The moderate predictive accuracy for the total score may result from challenges in aggregating predictions across components, as the total DGI score combines both healthy and unhealthy dietary components. For instance, discrepancies in predicting high-variability components, such as vegetable or discretionary food intake, could disproportionately influence the total score. Future research may require modification to diet quality measures that capture healthy and unhealthy components separately. Finally, while SHAP values can reveal feature importance made by the ML models from the data, they do not reflect causal relationship between contextual factors and food group consumption.

### Future research

This study demonstrated that ML can effectively predict food consumption at EOs based on contextual factors, laying a foundation for integrating ML models into just-in-time adaptive interventions (JITAIs) to improve diet quality. JITAIs are designed to respond to an individual’s evolving status and context, delivering tailored support at key moments [17]. The successful design of JITAIs requires identifying key components, including distal outcomes (e.g., adherence to dietary guidelines), proximal outcomes (e.g., increasing vegetable intake per EO), decision points (e.g., moments of high-risk for poor dietary choices), and tailoring variables (e.g., cooking confidence, perceived time scarcity).By leveraging the predictive insights from ML, JITAIs could deliver timely, personalised prompts to support healthier choices. For example, when examining all EOs for participant ID 161 over a single day, proximity and access to food emerged as the top factors of vegetable intake. This suggests that on days when access is limited, a timely prompt the day before, such as a reminder to meal prep, may help improve vegetable consumption. Alternatively, the JITAIs could deliver personalised dietary suggestions (e.g., eat more vegetables or order some) when the user is detected eating outside alone, using data from wearable or lifelogging devices. The ability to adapt interventions dynamically aligns with the incremental and consistent behavioural changes needed to achieve and sustain desirable dietary patterns, mitigating long-term health risks [64].

Future research should also build on this work by conducting longitudinal studies and more diverse population to capture the evolving nature of dietary behaviours and test the stability of ML-predicted factors over time. Such studies could also evaluate the effectiveness of JITAI-based interventions in promoting diet quality. Expanding research to include more diverse populations (representing varying cultural, socioeconomic, and age profiles) will be essential to ensure the broad applicability and equity of these interventions.

Beyond individual-level applications, ML has the potential to simulate the impact of modifying contextual variables at a population level. For instance, models could emulate scenarios such as increasing the availability of healthy foods, reducing the cost of fruits and vegetables, or enhancing the convenience of nutritious meal options. These simulations could provide valuable insights for policymakers, helping estimate the potential effects of policy interventions on population dietary patterns and health outcomes [65, 66].

## Conclusion

This study demonstrates the potential of ML models to predict food group consumption with high accuracy and diet quality with moderate accuracy, offering valuable insights into dietary behaviours at both individuals and EO. These findings underscore the utility of ML in addressing challenges in personalised nutrition, particularly in tailoring interventions to specific needs of individuals. Future research should expand on these findings by exploring the dynamic nature of dietary behaviours through longitudinal studies, evaluating the effectiveness of JITAI-based interventions, and testing their applicability across diverse populations.

## Supplementary Information

Supplementary Material 1.

Supplementary Material 2.

Supplementary Material 3.

Supplementary Material 4.

Supplementary Material 5.

## Data Availability

No datasets were generated or analysed during the current study.

## Abbreviations

EO: Eating occasion
EO: Level factors–eating occasion–level factors
ML: Machine learning
EMA: Ecological momentary assessment
ADG: Australian dietary guidelines
DGI: Dietary guideline index
SGBDT: Stochastic gradient boosting decision tree
RF: Random forest
RMSE: Root mean squared error
MAE: Mean absolute error
SHAP: Shapley additive explanations

## References

[1] Afshin A, Sur PJ, Fay KA, Cornaby L, Ferrara G, Salama JS, et al. Health effects of dietary risks in 195 countries, 1990–2017: a systematic analysis for the global burden of disease study 2017. Lancet. 2019;393(10184):1958–72. 30954305 10.1016/S0140-6736(19)30041-8 PMC6899507

[2] Dong C, Bu X, Liu J, Wei L, Ma A, Wang T. Cardiovascular disease burden attributable to dietary risk factors from 1990 to 2019: A systematic analysis of the global burden of disease study. Nutr Metabolism Cardiovasc Dis. 2022;32(4):897–907. 10.1016/j.numecd.2021.11.012 35067445

[3] Feigin VL, Stark BA, Johnson CO, Roth GA, Bisignano C, Abady GG, et al. Global, regional, and National burden of stroke and its risk factors, 1990–2019: a systematic analysis for the global burden of disease study 2019. Lancet Neurol. 2021;20(10):795–820. 34487721 10.1016/S1474-4422(21)00252-0 PMC8443449

[4] World Health Organization. Noncommunicable diseases. 2023. Available from: https://www.who.int/news-room/fact-sheets/detail/noncommunicable-diseases#:~:text=The%20four%20main%20types%20of%20noncommunicable%20diseases%20are%20cardiovascular%20diseases#:~:text=The%20four%20main%20types%20of%20noncommunicable%20diseases%20are%20cardiovascular%20diseases.

[5] Australian Bureau of Statistics. Dietary behaviour. 2023. Available from: https://www.abs.gov.au/statistics/health/health-conditions-and-risks/dietary-behaviour/latest-release.

[6] Thorpe MG, Kestin M, Riddell LJ, Keast RS, McNaughton SA. Diet quality in young adults and its association with food-related behaviours. Public Health Nutr. 2014;17(8):1767–75. 23866858 10.1017/S1368980013001924 PMC10282490

[7] McNaughton SA, Pendergast FJ, Worsley A, Leech RM. Eating occasion situational factors and sugar-sweetened beverage consumption in young adults. Int J Behav Nutr Phys Act. 2020;17(1):71. 32493366 10.1186/s12966-020-00975-y PMC7271392

[8] Leech RM, Worsley A, Timperio A, McNaughton SA. Characterizing eating patterns: a comparison of eating occasion definitions. Am J Clin Nutr. 2015;102(5):1229–37. 26447152 10.3945/ajcn.115.114660

[9] Leech RM, Worsley A, Timperio A, McNaughton SA. Temporal eating patterns: a latent class analysis approach. Int J Behav Nutr Phys Act. 2017;14(1):3. 28061795 10.1186/s12966-016-0459-6 PMC5219683

[10] Leech RM, Timperio A, Livingstone KM, Worsley A, McNaughton SA. Temporal eating patterns: associations with nutrient intakes, diet quality, and measures of adiposity. Am J Clin Nutr. 2017;106(4):1121–30. 28814392 10.3945/ajcn.117.156588

[11] Sobal J, Bisogni CA, Jastran M, Mind. Brain Educ. 2014;8(1):6–12.

[12] Sleddens EF, Kroeze W, Kohl LF, Bolten LM, Velema E, Kaspers P, et al. Correlates of dietary behavior in adults: an umbrella review. Nutr Rev. 2015;73(8):477–99. 26106126 10.1093/nutrit/nuv007 PMC4502713

[13] Tran NR, Leech RM, McNaughton SA. Contextual factors influence food intake at eating occasions in young adults: a mixed effect analysis. Appetite. 2024;203:107722. 10.1016/j.appet.2024.107722 39427723

[14] Symmank C, Mai R, Hoffmann S, Stok FM, Renner B, Lien N, et al. Predictors of food decision making: A systematic interdisciplinary mapping (SIM) review. Appetite. 2017;110:25–35. 27871944 10.1016/j.appet.2016.11.023

[15] Shiffman S, Stone AA, Hufford MR. Ecological momentary assessment. Ann Rev Clin Psychol. 2008;4(1):1–32. 10.1146/annurev.clinpsy.3.022806.091415 18509902

[16] Jinnette R, Narita A, Manning B, McNaughton SA, Mathers JC, Livingstone KM. Does personalized nutrition advice improve dietary intake in healthy adults? A systematic review of randomized controlled trials. Adv Nutr. 2021;12(3):657–69. 33313795 10.1093/advances/nmaa144 PMC8166555

[17] Nahum-Shani I, Smith SN, Spring BJ, Collins LM, Witkiewitz K, Tewari A, et al. Just-in-time adaptive interventions (JITAIs) in mobile health: key components and design principles for ongoing health behavior support. Ann Behav Med. 2018;52(6):446–62. 27663578 10.1007/s12160-016-9830-8 PMC5364076

[18] O’Hara C, Gibney ER. Meal pattern analysis in nutritional science: recent methods and findings. Adv Nutr. 2021;12(4):1365-78 . 10.1093/advances/nmaa175 PMC8321870 33460431

[19] Oliveira Chaves L, Gomes Domingos AL, Louzada Fernandes D, Ribeiro Cerqueira F, Siqueira-Batista R, Bressan J. Applicability of machine learning techniques in food intake assessment: A systematic review. Crit Rev Food Sci Nutr. 2023;63(7):902–19. 34323627 10.1080/10408398.2021.1956425

[20] Chiras D, Stamatopoulou M, Paraskevis N, Moustakidis S, Tzimitra-Kalogianni I, Kokkotis C. Explainable machine learning models for identification of Food-Related lifestyle factors in chicken meat consumption case in Northern Greece. BioMedInformatics. 2023;3(3):817–28.

[21] Tacardon ER, Ong AKS, Gumasing MJJ. Why are street foods consumed? A machine learning ensemble approach to assess consumption intention of street foods. Future Foods. 2023;8:100261.

[22] Mousavi H, Karandish M, Jamshidnezhad A, Hadianfard AM. Determining the effective factors in predicting diet adherence using an intelligent model. Sci Rep. 2022;12(1):12340. 35853992 10.1038/s41598-022-16680-8 PMC9296581

[23] Vandeputte J, Herold P, Kuslii M, Viappiani P, Muller L, Martin C, et al. Principles and validations of an artificial intelligence-based recommender system suggesting acceptable food changes. J Nutr. 2023;153(2):598–604. 36894251 10.1016/j.tjnut.2022.12.022

[24] Pendergast FJ, Livingstone KM, Worsley A, McNaughton SAJN. Examining the correlates of meal skipping in Australian young adults. Nutr J. 2019;18(1):24. 10.1186/s12937-019-0451-5 PMC6448264 30944008

[25] Pendergast FJ, Ridgers ND, Worsley A, McNaughton SA. Evaluation of a smartphone food diary application using objectively measured energy expenditure. Int J Behav Nutr Phys Activity. 2017;14(1):1–10. 10.1186/s12966-017-0488-9 PMC5348892 28288657

[26] Lachat C, Hawwash D, Ocké MC, Berg C, Forsum E, Hörnell A, et al. Strengthening the reporting of observational studies in Epidemiology–nutritional epidemiology (STROBE-nut): an extension of the STROBE statement. Nutr Bull. 2016;41(3):240–51. 27587981 10.1111/nbu.12217 PMC4988500

[27] Food Standards Australia New Zealand. Food consumption data used in dietary exposure assessments. 2014. Available from: https://www.foodstandards.gov.au/science/exposure/Pages/foodconsumptiondatau4440.aspx.

[28] Australian Bureau of Statistics. Discretionary foods. 2014. Available from: https://www.abs.gov.au/ausstats/abs@.nsf/Lookup/BA1526F0D19FA21DCA257CD2001CA166?opendocument.

[29] Food Standards Australia New Zealand. Classification of foods and dietary supplements. 2021. Available from: https://www.foodstandards.gov.au/science/monitoringnutrients/ausnut/classificationofsupps/Pages/default.aspx.

[30] McNaughton SA, Ball K, Crawford D, Mishra GD. An index of diet and eating patterns is a valid measure of diet quality in an Australian population. J Nutr. 2008;138(1):86–93. 18156409 10.1093/jn/138.1.86

[31] Thorpe MG, Milte CM, Crawford D, McNaughton SA. A revised Australian dietary guideline index and its association with key sociodemographic factors, health behaviors and body mass index in peri-retirement aged adults. Nutrients. 2016;8(3):160. 26978399 10.3390/nu8030160 PMC4808888

[32] Livingstone KM, McNaughton SA. Diet quality is associated with obesity and hypertension in Australian adults: a cross sectional study. BMC Public Health. 2016;16(1):1–10. 27716133 10.1186/s12889-016-3714-5 PMC5045600

[33] Australian Government Department of Health. Australia’s Physical Activity and Sedentary Behaviour Guidelines and the Australian 24-Hour Movement Guidelines. 2019. Available from: https://www1.health.gov.au/internet/main/publishing.nsf/Content/health-pubhlth-strateg-phys-act-guidelines#npa1864.

[34] Maddison R, Mhurchu CN, Jiang Y, Vander Hoorn S, Rodgers A, Lawes CM, et al. International physical activity questionnaire (IPAQ) and new Zealand physical activity questionnaire (NZPAQ): a doubly labelled water validation. Int J Behav Nutr Phys Activity. 2007;4(1):1–9. 10.1186/1479-5868-4-62 PMC2219963 18053188

[35] Larson NI, Nelson MC, Neumark-Sztainer D, Story M, Hannan PJ. Making time for meals: meal structure and associations with dietary intake in young adults. J Am Diet Assoc. 2009;109(1):72–9. 19103325 10.1016/j.jada.2008.10.017

[36] Smith KJ, McNaughton SA, Gall SL, Blizzard L, Dwyer T, Venn AJ. Involvement of young Australian adults in meal preparation: cross-sectional associations with abdominal obesity and body mass index. J Am Diet Assoc. 2011;111(8):1187–91. 21802565 10.1016/j.jada.2011.05.011

[37] Larson NI, Perry CL, Story M, Neumark-Sztainer D. Food Preparation by young adults is associated with better diet quality. J Am Diet Assoc. 2006;106(12):2001–7. 17126631 10.1016/j.jada.2006.09.008

[38] Sallis JF, Pinski RB, Grossman RM, Patterson TL, Nader PR. The development of self-efficacy scales for healthrelated diet and exercise behaviors. Health Educ Res. 1988;3(3):283–92.

[39] Health Education Authority. The HEA health and lifestyle survey: a report on the secondary analysis of a National dataset of health-related knowledge, attitudes and behaviour. London: Health Education Authority; 1998.

[40] Hendrie GA, Cox DN, Coveney J. Validation of the general nutrition knowledge questionnaire in an Australian community sample. Nutr Dietetics. 2008;65(1):72–7.

[41] Sallis JF, Grossman RM, Pinski RB, Patterson TL, Nader PR. The development of scales to measure social support for diet and exercise behaviors. Prev Med. 1987;16(6):825–36. 3432232 10.1016/0091-7435(87)90022-3

[42] Saelens BE, Sallis JF, Black JB, Chen D. Neighborhood-based differences in physical activity: an environment scale evaluation. Am J Public Health. 2003;93(9):1552–8. 12948979 10.2105/ajph.93.9.1552 PMC1448009

[43] Australian Bureau of Statistics. Socio-Economic Indexes for Areas. 2018. Available from: https://www.abs.gov.au/websitedbs/censushome.nsf/home/seifa.

[44] Kotsiantis SB, Zaharakis I, Pintelas P. Supervised machine learning: A review of classification techniques. Emerg Artif Intell Appl Comput Eng. 2007;160(1):3–24.

[45] Hastie T, Rosset S, Zhu J, Zou H. Multi-class adaboost. Stat its Interface. 2009;2(3):349–60.

[46] Liaw A, Wiener M. Classification and regression by randomforest. R News. 2002;2(3):18–22.

[47] Kunsch HR. The jackknife and the bootstrap for general stationary observations. Annals Stat. 1989;17(3):1217–41.

[48] Oshiro TM, Baranauskas JA, editors. Root attribute behavior within a random forest. International Conference on Intelligent Data Engineering and Automated Learning; 2012: Springer.

[49] Friedman JH. Stochastic gradient boosting. Comput Stat Data Anal. 2002;38(4):367–78.

[50] Lundberg SM, Erion G, Chen H, DeGrave A, Prutkin JM, Nair B, et al. From local explanations to global Understanding with explainable AI for trees. Nat Mach Intell. 2020;2(1):56–67. 32607472 10.1038/s42256-019-0138-9 PMC7326367

[51] Team RC. R: A Language and Environment for Statistical Computing. 2023. Available from: https://www.R-project.org/.

[52] Tsolakidis D, Gymnopoulos LP, Dimitropoulos K, editors. Artificial Intelligence and Machine Learning Technologies for Personalized Nutrition: A Review. Informatics; 2024.

[53] Siega-Riz AM, Popkin BM, Carson T. Differences in food patterns at breakfast by sociodemographic characteristics among a nationally representative sample of adults in the united States. Prev Med. 2000;30(5):415–24. 10845751 10.1006/pmed.2000.0651

[54] Jaeger SR, Marshall DW, Dawson J. A quantitative characterisation of meals and their contexts in a sample of 25 to 49-year-old Spanish people. Appetite. 2009;52(2):318–27. 19059444 10.1016/j.appet.2008.11.004

[55] Mueller Loose S, Jaeger SR. Factors that influence beverage choices at meal times. An application of the food choice kaleidoscope framework. Appetite. 2012;59(3):826–36. 22940686 10.1016/j.appet.2012.08.023

[56] Horgan GW, Scalco A, Craig T, Whybrow S, Macdiarmid JI. Social, Temporal and situational influences on meat consumption in the UK population. Appetite. 2019;138:1–9. 30858068 10.1016/j.appet.2019.03.007

[57] Theodore Armand TP, Nfor KA, Kim J-I, Kim H-C. Applications of artificial intelligence, machine learning, and deep learning in nutrition: A systematic review. Nutrients. 2024;16(7):1073. 38613106 10.3390/nu16071073 PMC11013624

[58] Maurya A, Wable R, Shinde R, John S, Jadhav R, Dakshayani R, editors. Chronic kidney disease prediction and recommendation of suitable diet plan by using machine learning. 2019 International Conference on Nascent Technologies in Engineering (ICNTE); 2019: IEEE.

[59] Mogaveera D, Mathur V, Waghela S, editors. e-Health monitoring system with diet and fitness recommendation using machine learning. 2021 6th International Conference on Inventive Computation Technologies (ICICT); 2021: IEEE.

[60] Iwendi C, Khan S, Anajemba JH, Bashir AK, Noor F. Realizing an efficient IoMT-assisted patient diet recommendation system through machine learning model. IEEE Access. 2020;8:28462–74.

[61] Sookrah R, Dhowtal JD, Nagowah SD, editors. A DASH diet recommendation system for hypertensive patients using machine learning. 2019 7th international conference on information and communication technology (ICoICT); 2019: IEEE.

[62] Ruf A, Neubauer AB, Ebner-Priemer U, Reif A, Matura S. Studying dietary intake in daily life through multilevel two-part modelling: a novel analytical approach and its practical application. Int J Behav Nutr Phys Activity. 2021;18(1):1–14. 10.1186/s12966-021-01187-8 PMC8477527 34579744

[63] Belle V, Papantonis I. Principles and practice of explainable machine learning. Front Big Data. 2021;4:688969. 34278297 10.3389/fdata.2021.688969 PMC8281957

[64] Dunton GF. Sustaining health-protective behaviors such as physical activity and healthy eating. JAMA. 2018;320(7):639–40. 29852046 10.1001/jama.2018.6621 PMC7524543

[65] dos Santos BS, Steiner MTA, Fenerich AT, Lima RHP. Data mining and machine learning techniques applied to public health problems: A bibliometric analysis from 2009 to 2018. Comput Ind Eng. 2019;138:106120.

[66] Bodnar LM, Cartus AR, Kirkpatrick SI, Himes KP, Kennedy EH, Simhan HN, et al. Machine learning as a strategy to account for dietary synergy: an illustration based on dietary intake and adverse pregnancy outcomes. Am J Clin Nutr. 2020;111(6):1235–43. 32108865 10.1093/ajcn/nqaa027 PMC7266693

