# Supplementary material for: Predicting diet quality and food consumption at eating occasions using contextual factors: an application of machine learning models
Source: Int J Behav Nutr Phys Act. 2025 Nov 4;22:136. doi: 10.1186/s12966-025-01818-4 (PMC12584291; doi:10.1186/s12966-025-01818-4)
Supplement: Supplementary file 3 — Supplementary Material 3. [file 12966_2025_1818_MOESM3_ESM.docx]

**Additional file 3. Percentage of missing values at each variable**

| Variable Name | Count of missing values (%) |
| --- | --- |
| **Person-level contextual factors** | |
| **Person-level Intrapersonal characteristics** | |
| Age | 0/675 (0.00) |
| Sex | 0/675 (0.00) |
| Country of birth | 0/675 (0.00) |
| Gross average income (AUD) per week | 44/675 (6.52) |
| Level of education | 0/675 (0.00) |
| Smoking status | 0/675 (0.00) |
| Physical Activity | 0/675 (0.00) |
| Meal Preparation behaviour score | 0/675 (0.00) |
| Food shopping behaviour score | 0/675 (0.00) |
| Self-efficacy score | 2/675 (0.30) |
| Cooking confidence score | 0/675 (0.00) |
| Perceived time scarcity | 0/675 (0.00) |
| Food choice barriers | 0/675 (0.00) |
| **Person-level Social-environmental factors** | |
| Social support from family | 1/675 (0.15) |
| Social support from friends | 0/675 (0.00) |
| **Person-level Physical-environmental factors** |  |
| Living situation | 0/675 (0.00) |
| Food nearness score | 2/675 (0.30) |
| Food availability score | 14/675 (2.07) |
| SEIFA | 2/675 (0.30) |
| **Eating-level contextual factors** |  |
| Presence of others | 0/11497 (0.00) |
| Activity at EO | 0/11497 (0.00) |
| Location of consumption | 0/11497 (0.00) |
| Location of food purchase | 0/11497 (0.00) |
| Preparation factor (Homemade or not) | 0/11497 (0.00) |

We used Little's missing completely at random (MCAR) test and Hawkins' test (R package MissMech) to assess whether the data was MCAR. The results showed a P-value of 0.278, indicating that the missing data was not systematically related to the observed values or any specific variable. Multiple imputation was performed using the R package mice, where missing values were filled with plausible estimates based on observed data. Five imputed datasets were created, with a maximum of 50 iterations for the imputation process. The Predictive Mean Matching (PMM) method was used to impute missing values by identifying observed values with similar predicted values. A seed value was set to ensure the process was reproducible, allowing the imputation to be repeated consistently.
