# Supplementary material for: Predicting diet quality and food consumption at eating occasions using contextual factors: an application of machine learning models
Source: Int J Behav Nutr Phys Act. 2025 Nov 4;22:136. doi: 10.1186/s12966-025-01818-4 (PMC12584291; doi:10.1186/s12966-025-01818-4)
Supplement: Supplementary file 4 — Supplementary Material 4. [file 12966_2025_1818_MOESM4_ESM.docx]

## Additional file 2. Contextual factors used for predicting food consumption and diet quality at eating occasions and its associated questionnaire measure and responses.

| **Factors** | **Measure** | **Original Responses** | **Collapse responses** | **Range** | **Ref** |
| --- | --- | --- | --- | --- | --- |
| **Person-level contextual factors** | | | | |  |
| **Intrapersonal factors** | | | | |  |
| Age | What is your date of birth? | 18–30 |  | Continuous |  |
| Sex | What gender do you identify as? | Male OR Female |  | 1-2 | - |
| Country of birth | In which country were you born? | Australia, UK, Italy, Greece, New Zealand, Vietnam, Other | Australia OR Outside Australia | 1-2 | - |
| Income | Gross average income (AUD) per week |  |  |  | - |
| Education | What is the highest qualification you have completed? | No formal qualification, Year 10 or equivalent, Year 12 or equivalent, Trade/apprenticeship, Certificate/diploma, University degree, Higher university degree | Year 12 or less OR Trade/apprentice or cert/dip OR Tertiary degree | 1–3 | - |
| Smoking status | Which of the following best describes your current smoking status? | I have never smoked before, I used to smoke, I now smoke occasionally, I now smoke regularly | Never smoked, current, past smoker | 1–3 | - |
| Self-efficacy | Seventeen-item scale to assess self-efficacy regarding health-related diet behaviours. | Not at all confident, slightly confident, moderately confident, very confident, extremely confident | Summed to form a total score, where 1=Not at all confident, 2=Slightly confident, 3=Moderately confident, 4=Very confident and 5=Extremely confident | 17–85 | (1) |
| Meal Preparation behaviour (food involvement) | On a scale of 1 (disagree strongly) to 7 (agree strongly), how much do you agree with the following statements?  1.I don’t think much about food each day.  2.Talking about what I ate or am going to eat is something I like to do.  3.Compared with other daily decisions, my food choices are not very important.  4.When I travel, one of the things I anticipate most is the eating the food there.  5.When I eat out, I don’t think or talk much about how the food tastes.  6.Cooking is not much fun.  7.I do most or all of the clean up after eating.  8.I enjoy cooking for others and myself.  9.I do not like to mix or chop food.  10.I do not wash dishes or clean the table.  11.I care whether or not the table is nicely set.  12.I do most or all of my own food shopping. | 1=Strongly disagree, 2, 3, 4, 5, 6, 7=Agree strongly | Summed to give a total score (items 1, 2, 4, 8, 9 and 11 were reverse scored) | 12–84 | (2) |
| Cooking confidence | Twenty-one item scale asking:  Which, if any, of these cooking techniques do you feel confident about using?  Which, if any, of these foods, do you feel confident about cooking? | Yes, No | Summed to give a total score, where No=0 and Yes=1 | 0–21 | (3) |
| Perceived time scarcity | How strongly do you agree with the following statements?  I am too busy to eat healthy foods.  I am too rushed in the morning to eat a healthy breakfast.  Eating healthy meals just takes too much time.  I do not have time to think about healthy eating. | Strongly disagree, disagree, agree, strongly agree | Summed to give a total score, where 1=Strongly disagree, 2=Disagree, 3=Agree and 4=Strongly agree | 4–16 | (2) |
| Food choice barriers | Which, if any, of these do you think limit the choice of food you buy?  ability to store food  limited cooking facilities or appliances  don’t know how to cook some foods  ability to carry and transport foods  food goes off before its eaten  difficult to get to shops with children  difficult to get to shops b/c of age or disability  finance/cost  time  another limitation  miscellaneous. | Yes, No | Summed to give a total score, where No=0 and Yes=1 | 0–11 | (2) |
| Physical activity | International Physical Activity Questionnaire—Short form (C) | Total minutes per week of vigorous intensity physical activity, and total minutes per week or moderate intensity physical activity | Meeting physical activity guidelines OR Not meeting physical activity guidelines | 1–2 | (4) |
| Person-level social-environmental factors | | | | | |
| Social support from family | During the past year, how often did members of your family (including spouse/partner):  eat healthy low-fat foods with you  encourage you to eat healthy low-fat foods  discourage you from eating unhealthy foods. | Original responses: Never, rarely, a few times, often, very often | Summed to give a total score, where 1=Never and 5=Very often. | 3–15 | (3, 5) |
| Social support from friends/colleagues | During the past year, how often did friends or work colleagues:  eat healthy low-fat foods with you  encourage you to eat healthy low-fat foods  discourage you from eating unhealthy foods. | Original responses: Never, rarely, a few times, often, very often | Summed to give a total score, where 1=Never and 5=Very often. | 3–15 | (3, 5) |
| Person-level physical environmental factors | | | | | |
| Proximity and access to food destinations | *About how long would it take to get from your home to the nearest stores or places listed below if you walked to them? | Original responses: 1–5 mins, 6–10 mins, 11–20 mins, 21–30 mins, 31 mins, don’t know  Within walking distance (≥15 mins), outside walking distance (<15 mins) | Summed and then averaged to give an overall score, where 1–5 min walk=5 and >30 min=1. | 16–63 | (6) |
| Food availability | The shops that serve my local community:  have a good supply of fresh fruit and vegetables  have good quality fruit and vegetables  sell dairy products that have a reduced-fat content. | Summed to give an overall score, where Neither agree or disagree=0, Strongly disagree =1, Disagree=2, Agree=3, Strongly agree=4. | Summed to give an overall score, where Neither agree or disagree=0, Strongly disagree =1, Disagree=2, Agree=3, Strongly agree=4. | 0–12 | (3) |
| Living situation | Which of the following best describes your housing or living situation? | Original responses: Living with parents/family, living by myself, living with partner/spouse, living with flatmates, living with children, other | Collapsed responses: Living with family or Living with flatmates/friends or Living alone | 1–3 | - |
| Area-level socio-economic position (SEIFA) | What is your current address? | SEIFA: 1–4=Low, 5–7=Medium, 8–10=High | Collapsed responses: Low (most disadvantaged) OR Medium OR High (least disadvantaged) | 1–3 | (7) |
| **Eating occasion-level environmental factors** | | | | |  |
| Place of consumption | What type of places were you at during this eating/ drinking occasion? | Home; Work; University; Family/ Friends Home; Restaurant; Coffee Shop/Café; Fast Food Venue; Sporting Venue; In Transit (car, train, bus); Other | Collapsed responses: Café/restaurant, Fast food venue, Home including family/friend’s home, in transit, Other/not stated, or Work/University. | 1-6 | (8) |
| Location of purchase | Where was this food or the main ingredients for the meal purchased from? | Large supermarket (Coles, Woolworths, IGA, Foodworks); Canteen (e.g. university/ school/ work canteen); Fast food/ takeaway shop; Restaurant/ café/ bar/ club; Local grocery store; Speciality shop (butcher, bakery, green grocer, delicatessen); Food market; Convenience store/ milkbar/ service station; Vending machine; Not sure; Other | Collapsed responses:  Convenience store/fast food outlet/vending, Restaurant/café/bar/club/canteen,  Supermarket/grocery store, or Other/not stated | 1-4 | (8) |
| **Eating occasion-level social contextual factors** | | | | | |
| Presence of others | Who were you with while eating/ drinking? | By myself; With Friends, With my partner, With my children; With other family members; With work colleagues; With other Uni students; Other | Collapsed responses:  Alone, With friends (which includes with friends; With work colleagues; With other Uni students), With other people (which includes with my partner, With my children; With other family members), or other/not stated | 1-4 | (8) |
| Activity at consumption | What were you doing while eating/ drinking? | Nothing else, just eating; Watching TV/movies/cinema; Using the computer; In transit (car, train, tram, bus); Talking/texting on the phone; Visiting or socialising with friends/family; Reading/ studying; Playing sports; Other | Collapsed responses:  Nothing, Screen-based activity (which includes Watching TV/movies/cinema; Using the computer; Talking/texting on the phone), Visiting family/friends, or Other/not stated (which includes Visiting or socialising with friends/family; Reading/ studying; Playing sports; Other) | 1-4 | (8) |

1. Sallis JF, Pinski RB, Grossman RM, Patterson TL, Nader PR. The development of self-efficacy scales for healthrelated diet and exercise behaviors. Health education research. 1988;3(3):283-92.

2. Larson NI, Nelson MC, Neumark-Sztainer D, Story M, Hannan PJ. Making time for meals: meal structure and associations with dietary intake in young adults. Journal of the American dietetic association. 2009;109(1):72-9.

3. Health Education Authority. The HEA health and lifestyle survey : a report on the secondary analysis of a national dataset of health-related knowledge, attitudes and behaviour. London : Health Education Authority; 1998.

4. Australian Government Department of Health. Australia's Physical Activity and Sedentary Behaviour Guidelines and the Australian 24-Hour Movement Guidelines 2019 [Available from: <https://www1.health.gov.au/internet/main/publishing.nsf/Content/health-pubhlth-strateg-phys-act-guidelines#npa1864>.

5. Sallis JF, Grossman RM, Pinski RB, Patterson TL, Nader PR. The development of scales to measure social support for diet and exercise behaviors. Preventive medicine. 1987;16(6):825-36.

6. Saelens BE, Sallis JF, Black JB, Chen D. Neighborhood-based differences in physical activity: an environment scale evaluation. American journal of public health. 2003;93(9):1552-8.

7. Australian Bureau of Statistics. Socio-Economic Indexes for Areas 2018 [Available from: <https://www.abs.gov.au/websitedbs/censushome.nsf/home/seifa>.

8. Pendergast FJ, Ridgers ND, Worsley A, McNaughton SA. Evaluation of a smartphone food diary application using objectively measured energy expenditure. International Journal of Behavioral Nutrition and Physical Activity. 2017;14(1):1-10.
