# Supplementary material for: Predicting diet quality and food consumption at eating occasions using contextual factors: an application of machine learning models
Source: Int J Behav Nutr Phys Act. 2025 Nov 4;22:136. doi: 10.1186/s12966-025-01818-4 (PMC12584291; doi:10.1186/s12966-025-01818-4)
Supplement: Supplementary file 5 — Supplementary Material 5. [file 12966_2025_1818_MOESM5_ESM.docx]

## Additional file 4. Mean Absolute SHAP values for instances at one eating occasion, one day, one individual, and all individuals.

***Local Feature Importance Example (continue) - Predicting Discretionary Food Intakes***

When investigating factor importance in predicting the occurrence of discretionary food consumption, the top five factors for a single eating occasion were meal preparation, place of consumption, age, activity at consumption, and cooking confidence. At the daily level, the top factors were physical activity, food barriers, presence of others at consumption, perceived time barriers, and meal preparation. At the individual level, the top five factors influencing the prediction of discretionary food consumption for participant ID 161 were country of birth, gender, food availability, place of consumption, and level of income (Additional file 4, Table 4.6).

When discretionary food consumption occurred, the top five factors influencing the amount consumed were the presence of others at consumption, place of purchase, self-efficacy, cooking confidence, and gender. At the daily level, the top factors were physical activity, smoking status, level of income, place of consumption, and gender. At the individual level, the top five factors for participant ID 161 were place of purchase, smoking status, place of consumption, food barriers, and social support from family (Additional file 4, Table 4.6).

***Case Study Example (continue) - Predicting Daily Diet Quality***

For predicting the daily diet quality of participant ID 161, the top five factors for one observation day were level of income, level of education, SEIFA, place of consumption, and smoking status. When assessing overall factor importance for predicting the diet quality of participant ID 161, the top five factors were age, food barriers, food availability, perceived time barriers, and self-efficacy (Additional file 4, Table 4.7).

### Table A4.1 Mean Absolute SHAP values to assess predictors impact for predicting fruit consumption. (Results presented were randomly chosen. Values extracted from eating occasion = lunch, Day = 3, ID = 161, All = All IDs)).

| **Contextual factors** | **Eating Occasion** | **Day** | **Individual** | **All** |
| --- | --- | --- | --- | --- |
| Person-level: Intrapersonal factors |  |  |  |  |
| Age | 0.0058 | 0.0020 | 0.0014 | 0.0003 |
| Sex | 0.0003 | 0.0010 | 0.0015 | 0.0004 |
| Country of birth | 0.0005 | 0.0023 | 0.0005 | 0.0003 |
| Income | 0.0001 | 0.0032 | 0.0021 | 0.0004 |
| Education | 0.0023 | 0.0023 | 0.0030 | 0.0003 |
| Smoking status | 0.0024 | 0.0031 | 0.0044 | 0.0003 |
| Self-efficacy | 0.0014 | 0.0011 | 0.0009 | 0.0005 |
| Meal Preparation behaviour (food involvement) | 0.0005 | 0.0003 | 0.0026 | 0.0003 |
| Cooking confidence | 0.0114 | 0.0004 | 0.0021 | 0.0005 |
| Perceived time scarcity | 0.0064 | 0.0004 | 0.0015 | 0.0004 |
| Food choice barriers | 0.0014 | 0.0013 | 0.0006 | 0.0005 |
| Physical activity | 0.0026 | 0.0056 | 0.0011 | 0.0004 |
| Person-level: Socio-environmental factors |  |  |  |  |
| Social support from family | 0.0028 | 0.0033 | 0.0040 | 0.0005 |
| Social support from friends/colleagues | 0.0025 | 0.0011 | 0.0021 | 0.0003 |
| Person-level: Physical environmental factors |  |  |  |  |
| Proximity and access to food destinations | 0.0031 | 0.0005 | 0.0028 | 0.0003 |
| Food availability | 0.0032 | 0.0025 | 0.0001 | 0.0005 |
| Living situation | 0.0012 | 0.0045 | 0.0004 | 0.0004 |
| Area-level socio-economic position (SEIFA) | 0.0031 | 0.0028 | 0.0030 | 0.0003 |
| Eating occasion-level: Environmental factors |  |  |  |  |
| Place of consumption | 0.0113 | 0.0023 | 0.0027 | 0.0004 |
| Location of purchase | 0.0143 | 0.0026 | 0.0035 | 0.0004 |
| Eating occasion-level: Social factors |  |  |  |  |
| Presence of others | 0.0138 | 0.0010 | 0.0015 | 0.0004 |
| Activity at consumption | 0.0005 | 0.0006 | 0.0010 | 0.0004 |

### Table A4.2 Mean Absolute SHAP values to assess predictors impact for predicting vegetable consumption (Results presented were randomly chosen. Values extracted from eating occasion = lunch, Day = 3, ID = 161, All = All IDs)).

| **Contextual factors** | **Eating Occasion** | **Day** | **Individual** | **All** |
| --- | --- | --- | --- | --- |
| Person-level: Intrapersonal factors |  |  |  |  |
| Age | 0.0110 | 0.0063 | 0.0099 | 0.0033 |
| Sex | 0.0069 | 0.0111 | 0.0013 | 0.0033 |
| Country of birth | 0.0039 | 0.0088 | 0.0050 | 0.0031 |
| Income | 0.0069 | 0.0121 | 0.0060 | 0.0035 |
| Education | 0.0065 | 0.0063 | 0.0040 | 0.0032 |
| Smoking status | 0.0055 | 0.0136 | 0.0095 | 0.0031 |
| Self-efficacy | 0.0011 | 0.0006 | 0.0047 | 0.0030 |
| Meal Preparation behaviour (food involvement) | 0.0115 | 0.0092 | 0.0068 | 0.0030 |
| Cooking confidence | 0.0047 | 0.0001 | 0.0008 | 0.0031 |
| Perceived time scarcity | 0.0107 | 0.0125 | 0.0053 | 0.0033 |
| Food choice barriers | 0.0096 | 0.0033 | 0.0083 | 0.0030 |
| Physical activity | 0.0053 | 0.0074 | 0.0037 | 0.0032 |
| Person-level: Socio-environmental factors |  |  |  |  |
| Social support from family | 0.0077 | 0.0040 | 0.0074 | 0.0034 |
| Social support from friends/colleagues | 0.0029 | 0.0027 | 0.0040 | 0.0033 |
| Person-level: Physical environmental factors |  |  |  |  |
| Proximity and access to food destinations | 0.0156 | 0.0248 | 0.0054 | 0.0034 |
| Food availability | 0.0029 | 0.0034 | 0.0032 | 0.0030 |
| Living situation | 0.0010 | 0.0041 | 0.0008 | 0.0031 |
| Area-level socio-economic position (SEIFA) | 0.0158 | 0.0071 | 0.0029 | 0.0035 |
| Eating occasion-level: Environmental factors |  |  |  |  |
| Place of consumption | 0.0121 | 0.0121 | 0.0116 | 0.0031 |
| Location of purchase | 0.0060 | 0.0156 | 0.0094 | 0.0032 |
| Eating occasion-level: Social factors |  |  |  |  |
| Presence of others | 0.0739 | 0.0014 | 0.0052 | 0.0031 |
| Activity at consumption | 0.0020 | 0.0035 | 0.0050 | 0.0032 |

### Figure A4.1 Mean Absolute SHAP values for dairy intakes.


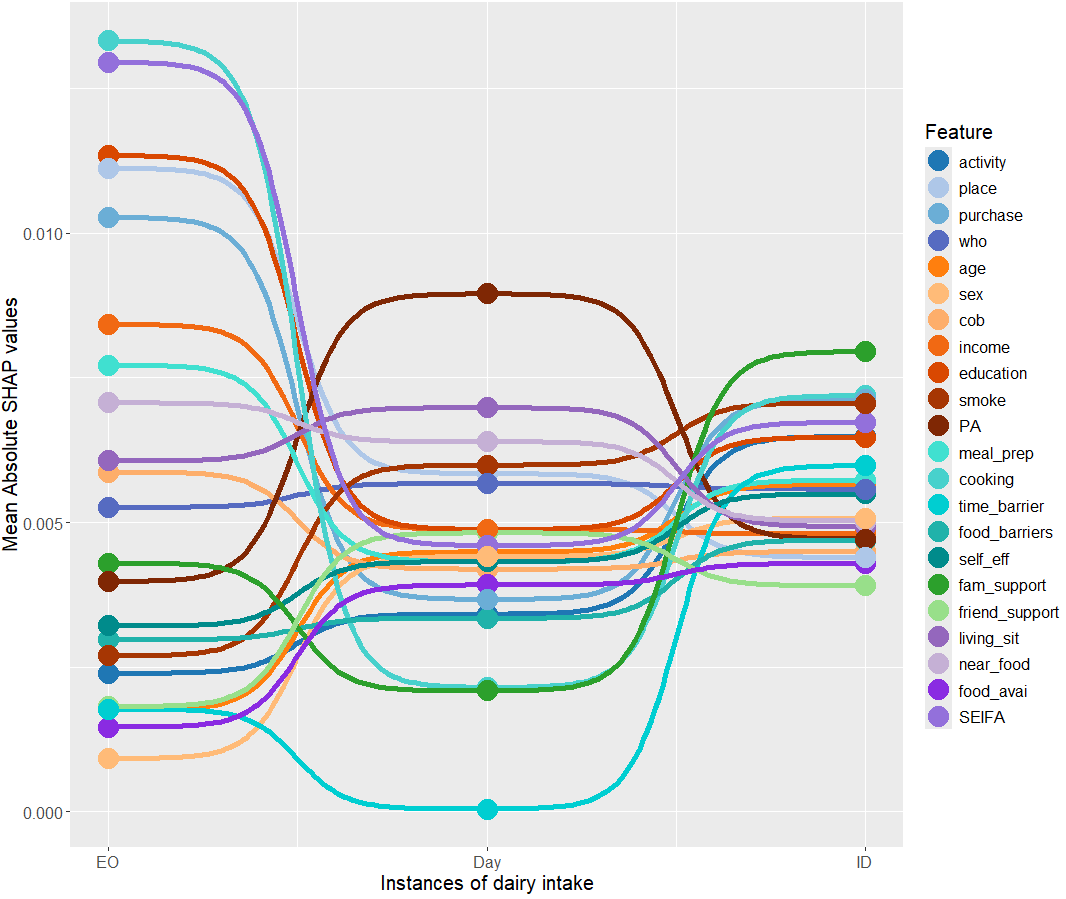


### Table A4.3 Mean Absolute SHAP values to assess predictors impact for predicting dairy and its alternative consumption.

| **Contextual factors** | **Eating Occasion** | **Day** | **Individual** | **All** |
| --- | --- | --- | --- | --- |
| Person-level: Intrapersonal factors |  |  |  |  |
| Age | 0.0018 | 0.0045 | 0.0057 | 0.0010 |
| Sex | 0.0009 | 0.0044 | 0.0051 | 0.0009 |
| Country of birth | 0.0059 | 0.0042 | 0.0045 | 0.0011 |
| Income | 0.0084 | 0.0049 | 0.0048 | 0.0010 |
| Education | 0.0113 | 0.0049 | 0.0065 | 0.0012 |
| Smoking status | 0.0027 | 0.0060 | 0.0071 | 0.0010 |
| Self-efficacy | 0.0032 | 0.0043 | 0.0055 | 0.0009 |
| Meal Preparation behaviour (food involvement) | 0.0077 | 0.0043 | 0.0057 | 0.0010 |
| Cooking confidence | 0.0133 | 0.0021 | 0.0072 | 0.0010 |
| Perceived time scarcity | 0.0018 | 0.0000 | 0.0060 | 0.0018 |
| Food choice barriers | 0.0030 | 0.0033 | 0.0047 | 0.0011 |
| Physical activity | 0.0040 | 0.0090 | 0.0047 | 0.0011 |
| Person-level: Socio-environmental factors |  |  |  |  |
| Social support from family | 0.0015 | 0.0039 | 0.0043 | 0.0011 |
| Social support from friends/colleagues | 0.0018 | 0.0048 | 0.0039 | 0.0011 |
| Person-level: Physical environmental factors |  |  |  |  |
| Proximity and access to food destinations | 0.0071 | 0.0064 | 0.0050 | 0.0010 |
| Food availability | 0.0015 | 0.0039 | 0.0043 | 0.0011 |
| Living situation | 0.0061 | 0.0070 | 0.0049 | 0.0010 |
| Area-level socio-economic position (SEIFA) | 0.0130 | 0.0046 | 0.0067 | 0.0009 |
| Eating occasion-level: Environmental factors |  |  |  |  |
| Place of consumption | 0.0111 | 0.0058 | 0.0044 | 0.0010 |
| Location of purchase | 0.0103 | 0.0037 | 0.0071 | 0.0010 |
| Eating occasion-level: Social factors |  |  |  |  |
| Presence of others | 0.0053 | 0.0057 | 0.0056 | 0.0009 |
| Activity at consumption | 0.0024 | 0.0034 | 0.0065 | 0.0010 |

### Figure A3.2 Mean Absolute SHAP values for meats and alternative intakes.


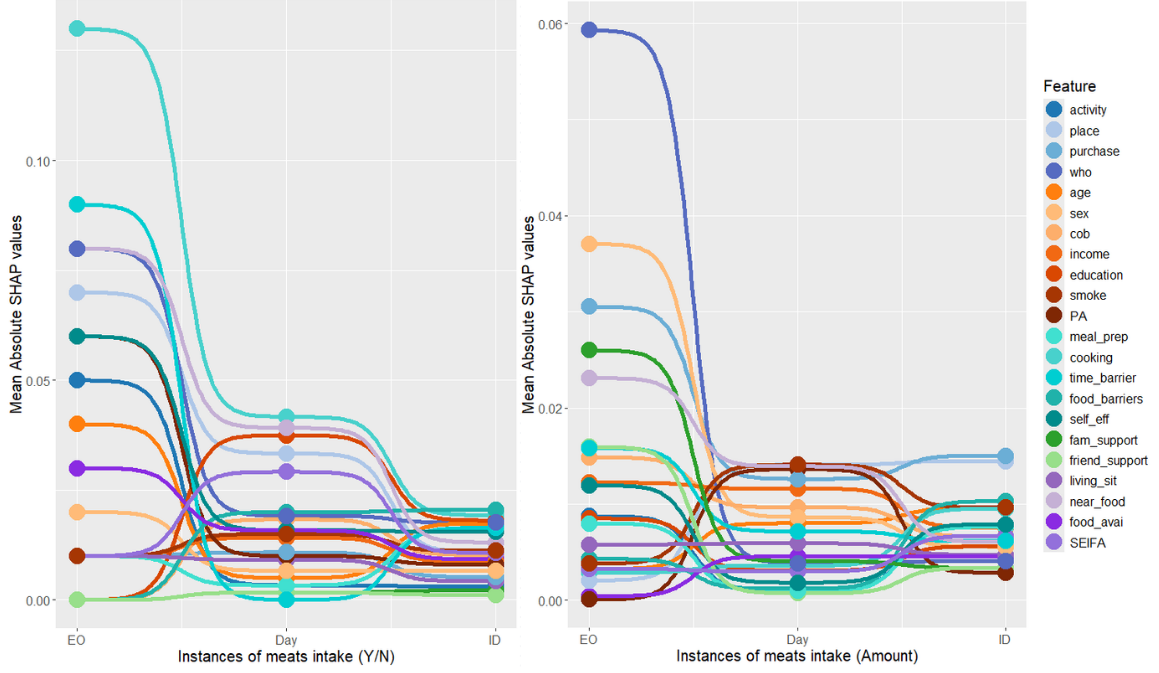


### Table A4.4 Mean Absolute SHAP values to assess predictors impact for predicting meats and its alternatives consumption.

|  | | *Mean Absolute SHAP values* | | | | | | | |
| --- | --- | --- | --- | --- | --- | --- | --- | --- | --- |
| Contextual factors | | **Binary outcome (consumption y/n)** | | | | **Regression outcome (amount)** | | | |
| Type | **Feature name** | **Eating Occasion** | **Day** | **Individual** | **All** | **Eating Occasion** | **Day** | **Individual** | **All** |
| Person-level: Intrapersonal factors | Age | 0.0400 | 0.0050 | 0.0174 | 0.0004 | 0.0033 | 0.0080 | 0.0096 | 0.0011 |
|  | Sex | 0.0200 | 0.0067 | 0.0067 | 0.0004 | 0.0371 | 0.0087 | 0.0056 | 0.0012 |
|  | Country of birth | 0.0000 | 0.0183 | 0.0102 | 0.0008 | 0.0149 | 0.0097 | 0.0067 | 0.0012 |
|  | Income | 0.0100 | 0.0142 | 0.0088 | 0.0002 | 0.0122 | 0.0116 | 0.0076 | 0.0013 |
|  | Education | 0.0000 | 0.0375 | 0.0181 | 0.0008 | 0.0085 | 0.0031 | 0.0056 | 0.0012 |
|  | Smoking status | 0.0100 | 0.0150 | 0.0112 | 0.0003 | 0.0038 | 0.0141 | 0.0097 | 0.0014 |
|  | Self-efficacy | 0.0600 | 0.0158 | 0.0155 | 0.0006 | 0.0120 | 0.0018 | 0.0079 | 0.0013 |
|  | Meal Preparation behaviour (food involvement) | 0.0100 | 0.0033 | 0.0162 | 0.0008 | 0.0080 | 0.0010 | 0.0076 | 0.0012 |
|  | Cooking confidence | 0.1300 | 0.0417 | 0.0193 | 0.0010 | 0.0029 | 0.0036 | 0.0095 | 0.0013 |
|  | Perceived time scarcity | 0.0900 | 0.0000 | 0.0164 | 0.0001 | 0.0158 | 0.0072 | 0.0062 | 0.0011 |
|  | Food choice barriers | 0.0000 | 0.0200 | 0.0205 | 0.0009 | 0.0043 | 0.0012 | 0.0103 | 0.0013 |
|  | Physical activity | 0.0600 | 0.0100 | 0.0081 | 0.0002 | 0.0001 | 0.0137 | 0.0029 | 0.0013 |
| Person-level: Socio-environmental factors | Social support from family | 0.0000 | 0.0017 | 0.0021 | 0.0000 | 0.0260 | 0.0041 | 0.0034 | 0.0011 |
|  | Social support from friends/colleagues | 0.0000 | 0.0017 | 0.0012 | 0.0004 | 0.0160 | 0.0007 | 0.0034 | 0.0011 |
| Person-level: Physical environmental factors | Proximity and access to food destinations | 0.0800 | 0.0392 | 0.0131 | 0.0009 | 0.0231 | 0.0139 | 0.0061 | 0.0011 |
|  | Food availability | 0.0300 | 0.0158 | 0.0093 | 0.0006 | 0.0004 | 0.0046 | 0.0046 | 0.0013 |
|  | Living situation | 0.0100 | 0.0092 | 0.0043 | 0.0005 | 0.0058 | 0.0059 | 0.0047 | 0.0011 |
|  | Area-level socio-economic position (SEIFA) | 0.0100 | 0.0292 | 0.0107 | 0.0002 | 0.0033 | 0.0030 | 0.0067 | 0.0011 |
| Eating occasion-level: Environmental factors | Place of consumption | 0.0700 | 0.0333 | 0.0110 | 0.0002 | 0.0021 | 0.0141 | 0.0145 | 0.0012 |
|  | Location of purchase | 0.0100 | 0.0108 | 0.0052 | 0.0002 | 0.0306 | 0.0126 | 0.0151 | 0.0014 |
| Eating occasion-level: Social factors | Presence of others | 0.0800 | 0.0192 | 0.0176 | 0.0001 | 0.0593 | 0.0039 | 0.0041 | 0.0012 |
|  | Activity at consumption | 0.0500 | 0.0033 | 0.0031 | 0.0004 | 0.0088 | 0.0011 | 0.0078 | 0.0012 |

### Figure A4.3 Mean Absolute SHAP values for grains and alternative intakes.


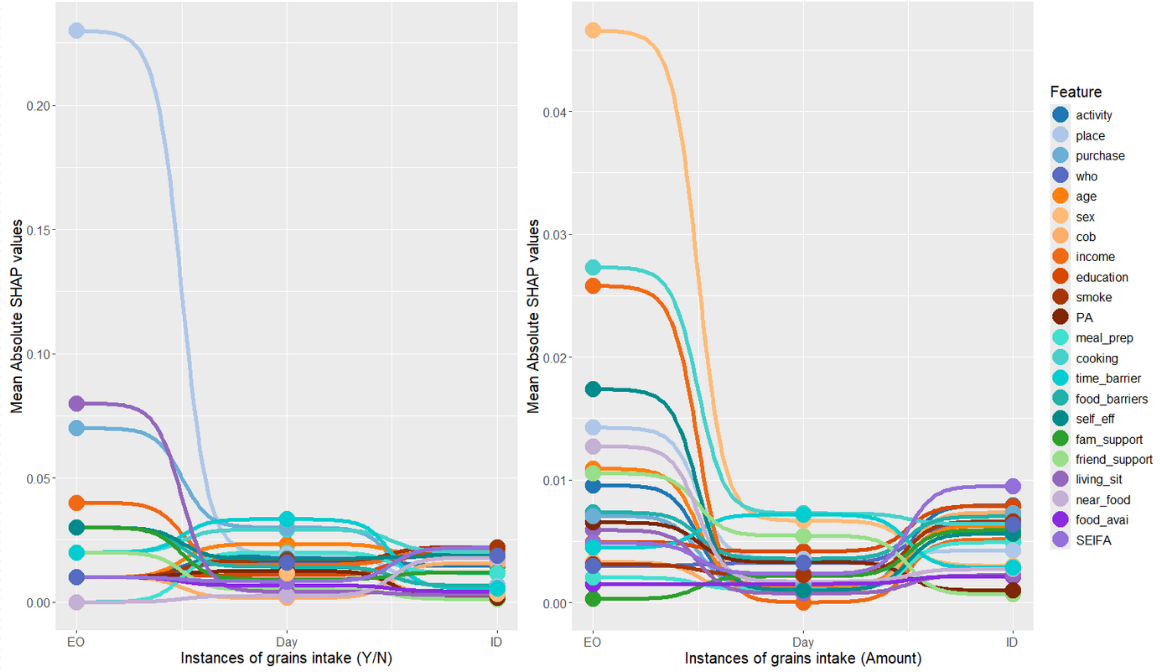


### Table A4.5 Mean Absolute SHAP values to assess predictors impact for predicting grain consumption.

|  | | *Mean Absolute SHAP values* | | | | | | | |
| --- | --- | --- | --- | --- | --- | --- | --- | --- | --- |
| Features/Predictors | | **Binary outcome (consumption y/n)** | | | | **Regression outcome (amount)** | | | |
| Type | **Feature name** | **Eating Occasion** | **Day** | **Individual** | **All** | **Eating Occasion** | **Day** | **Individual** | **All** |
| Person-level: Intrapersonal factors | Age | 0.0100 | 0.0233 | 0.0181 | 0.0007 | 0.0109 | 0.0014 | 0.0063 | 0.0007 |
|  | Sex | 0.0100 | 0.0117 | 0.0033 | 0.0007 | 0.0466 | 0.0067 | 0.0030 | 0.0006 |
|  | Country of birth | 0.0100 | 0.0017 | 0.0157 | 0.0023 | 0.0033 | 0.0009 | 0.0073 | 0.0007 |
|  | Income | 0.0400 | 0.0150 | 0.0205 | 0.0016 | 0.0258 | 0.0000 | 0.0051 | 0.0005 |
|  | Education | 0.0100 | 0.0108 | 0.0202 | 0.0024 | 0.0050 | 0.0042 | 0.0079 | 0.0006 |
|  | Smoking status | 0.0100 | 0.0167 | 0.0221 | 0.0015 | 0.0031 | 0.0023 | 0.0066 | 0.0006 |
|  | Self-efficacy | 0.0300 | 0.0175 | 0.0186 | 0.0015 | 0.0174 | 0.0010 | 0.0056 | 0.0007 |
|  | Meal Preparation behaviour (food involvement) | 0.0000 | 0.0200 | 0.0117 | 0.0013 | 0.0020 | 0.0011 | 0.0049 | 0.0007 |
|  | Cooking confidence | 0.0200 | 0.0292 | 0.0202 | 0.0010 | 0.0273 | 0.0073 | 0.0064 | 0.0005 |
|  | Perceived time scarcity | 0.0200 | 0.0333 | 0.0055 | 0.0011 | 0.0045 | 0.0072 | 0.0028 | 0.0007 |
|  | Food choice barriers | 0.0300 | 0.0142 | 0.0067 | 0.0009 | 0.0073 | 0.0036 | 0.0070 | 0.0008 |
|  | Physical activity | 0.0100 | 0.0125 | 0.0017 | 0.0014 | 0.0065 | 0.0033 | 0.0010 | 0.0006 |
| Person-level: Socio-environmental factors | Social support from family | 0.0300 | 0.0092 | 0.0119 | 0.0001 | 0.0003 | 0.0022 | 0.0059 | 0.0006 |
|  | Social support from friends/colleagues | 0.0200 | 0.0050 | 0.0012 | 0.0009 | 0.0105 | 0.0055 | 0.0007 | 0.0007 |
| Person-level: Physical environmental factors | Proximity and access to food destinations | 0.0000 | 0.0025 | 0.0179 | 0.0015 | 0.0127 | 0.0017 | 0.0027 | 0.0007 |
|  | Food availability | 0.0100 | 0.0067 | 0.0043 | 0.0023 | 0.0015 | 0.0015 | 0.0021 | 0.0006 |
|  | Living situation | 0.0800 | 0.0042 | 0.0029 | 0.0011 | 0.0059 | 0.0008 | 0.0022 | 0.0008 |
|  | Area-level socio-economic position (SEIFA) | 0.0100 | 0.0083 | 0.0219 | 0.0016 | 0.0049 | 0.0023 | 0.0095 | 0.0007 |
| Eating occasion-level: Environmental factors | Place of consumption | 0.2300 | 0.0192 | 0.0062 | 0.0019 | 0.0143 | 0.0036 | 0.0042 | 0.0006 |
|  | Location of purchase | 0.0700 | 0.0300 | 0.0057 | 0.0022 | 0.0071 | 0.0012 | 0.0073 | 0.0007 |
| Eating occasion-level: Social factors | Presence of others | 0.0100 | 0.0158 | 0.0186 | 0.0017 | 0.0030 | 0.0032 | 0.0064 | 0.0006 |
|  | Activity at consumption | 0.0100 | 0.0183 | 0.0145 | 0.0030 | 0.0096 | 0.0012 | 0.0079 | 0.0006 |

### Figure A4.4 Feature importance based on mean absolute SHAP values for best predicting model for discretionary foods intake (Hurdle Random Forest) at eating occasion. Results presented were randomly chosen to show changes in importance of features/predictors at different level of instance (random state = 30820); EO included one eating occasion, Day included all eating occasion in one day, and ID included all eating occasions recorded by one individual (ID = 161). The mean absolute value of each feature over all instances (all eating occasions recorded from all individuals) is not presented but reported in Additional file 3. A feature/predictor with a higher mean absolute SHAP values contributes more significantly to the prediction on average.


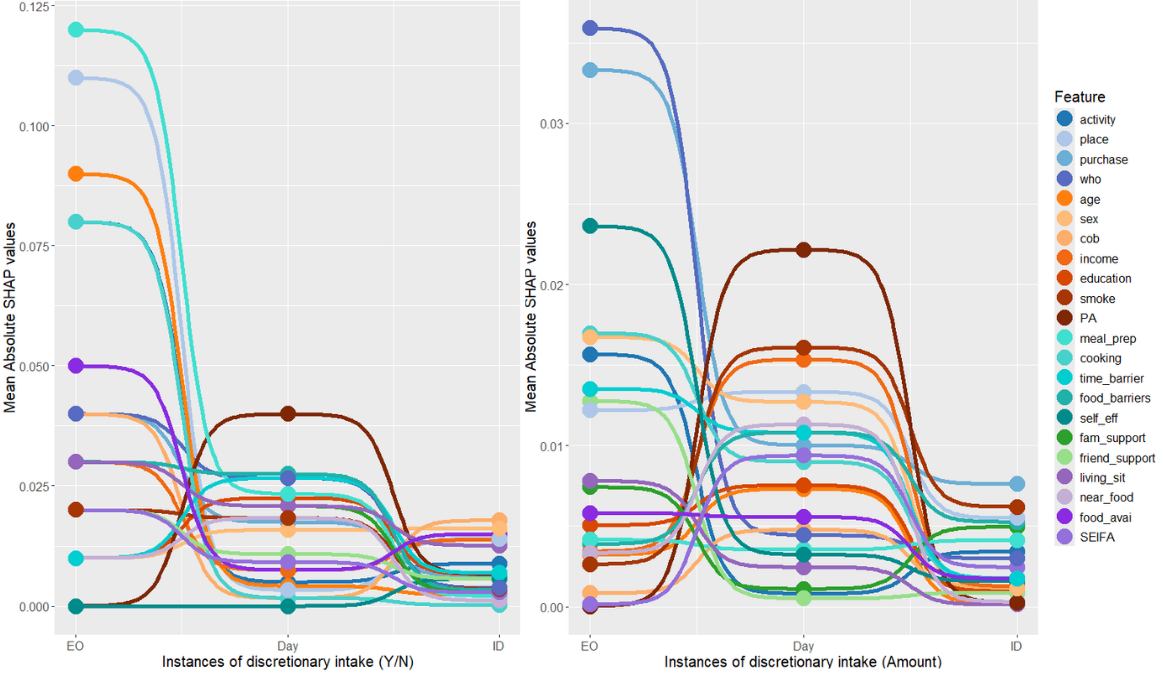


### Table A4.6 Mean Absolute SHAP values to assess predictors impact for predicting discretionary foods consumption.

|  | | *Mean Absolute SHAP values* | | | | | | | |
| --- | --- | --- | --- | --- | --- | --- | --- | --- | --- |
| Features/Predictors | | **Binary outcome (consumption y/n)** | | | | **Regression outcome (amount)** | | | |
| Type | **Feature name** | **Eating Occasion** | **Day** | **Individual** | **All** | **Eating Occasion** | **Day** | **Individual** | **All** |
| Person-level: Intrapersonal factors | Age | 0.0900 | 0.0042 | 0.0024 | 0.0014 | 0.0032 | 0.0073 | 0.0002 | 0.0036 |
|  | Sex | 0.0100 | 0.0158 | 0.0162 | 0.0011 | 0.0167 | 0.0127 | 0.0012 | 0.0033 |
|  | Country of birth | 0.0400 | 0.0017 | 0.0179 | 0.0019 | 0.0008 | 0.0048 | 0.0012 | 0.0033 |
|  | Income | 0.0300 | 0.0075 | 0.0138 | 0.0009 | 0.0034 | 0.0154 | 0.0013 | 0.0032 |
|  | Education | 0.0100 | 0.0225 | 0.0067 | 0.0022 | 0.0051 | 0.0075 | 0.0010 | 0.0034 |
|  | Smoking status | 0.0200 | 0.0183 | 0.0036 | 0.0019 | 0.0026 | 0.0161 | 0.0062 | 0.0035 |
|  | Self-efficacy | 0.0000 | 0.0000 | 0.0057 | 0.0015 | 0.0236 | 0.0032 | 0.0016 | 0.0035 |
|  | Meal Preparation behaviour (food involvement) | 0.1200 | 0.0233 | 0.0021 | 0.0014 | 0.0042 | 0.0036 | 0.0041 | 0.0035 |
|  | Cooking confidence | 0.0800 | 0.0017 | 0.0002 | 0.0012 | 0.0170 | 0.0090 | 0.0018 | 0.0030 |
|  | Perceived time scarcity | 0.0100 | 0.0267 | 0.0069 | 0.0014 | 0.0135 | 0.0108 | 0.0018 | 0.0037 |
|  | Food choice barriers | 0.0300 | 0.0275 | 0.0033 | 0.0014 | 0.0039 | 0.0108 | 0.0053 | 0.0034 |
|  | Physical activity | 0.0000 | 0.0400 | 0.0067 | 0.0007 | 0.0000 | 0.0222 | 0.0003 | 0.0033 |
| Person-level: Socio-environmental factors | Social support from family | 0.0300 | 0.0208 | 0.0029 | 0.0012 | 0.0074 | 0.0011 | 0.0050 | 0.0036 |
|  | Social support from friends/colleagues | 0.0200 | 0.0108 | 0.0057 | 0.0023 | 0.0128 | 0.0005 | 0.0009 | 0.0034 |
| Person-level: Physical environmental factors | Proximity and access to food destinations | 0.0100 | 0.0183 | 0.0012 | 0.0021 | 0.0034 | 0.0113 | 0.0003 | 0.0036 |
|  | Food availability | 0.0500 | 0.0075 | 0.0150 | 0.0008 | 0.0058 | 0.0056 | 0.0018 | 0.0032 |
|  | Living situation | 0.0300 | 0.0208 | 0.0126 | 0.0006 | 0.0078 | 0.0025 | 0.0001 | 0.0035 |
|  | Area-level socio-economic position (SEIFA) | 0.0200 | 0.0092 | 0.0029 | 0.0017 | 0.0002 | 0.0094 | 0.0025 | 0.0035 |
| Eating occasion-level: Environmental factors | Place of consumption | 0.1100 | 0.0033 | 0.0140 | 0.0018 | 0.0122 | 0.0133 | 0.0055 | 0.0037 |
|  | Location of purchase | 0.0400 | 0.0175 | 0.0062 | 0.0024 | 0.0333 | 0.0100 | 0.0076 | 0.0034 |
| Eating occasion-level: Social factors | Presence of others | 0.0400 | 0.0267 | 0.0038 | 0.0013 | 0.0359 | 0.0044 | 0.0030 | 0.0034 |
|  | Activity at consumption | 0.0800 | 0.0050 | 0.0088 | 0.0011 | 0.0157 | 0.0008 | 0.0034 | 0.0034 |

### Figure A4.5 Feature importance based on mean SHAP values for best predicting model for daily diet quality (Random Forest). Results presented were randomly chosen to show changes in importance of features/predictors at different level of instance (random state = 30820); Day indicates daily diet quality (DGI score), ID included every daily diet quality (DGI score) recorded from one individual (ID = 161), and All included all daily diet quality recorded from all individuals. The mean absolute value of each feature over all instances is presented in Additional file 3. A feature/predictor with a higher mean absolute SHAP values contributes more significantly to the prediction on average.


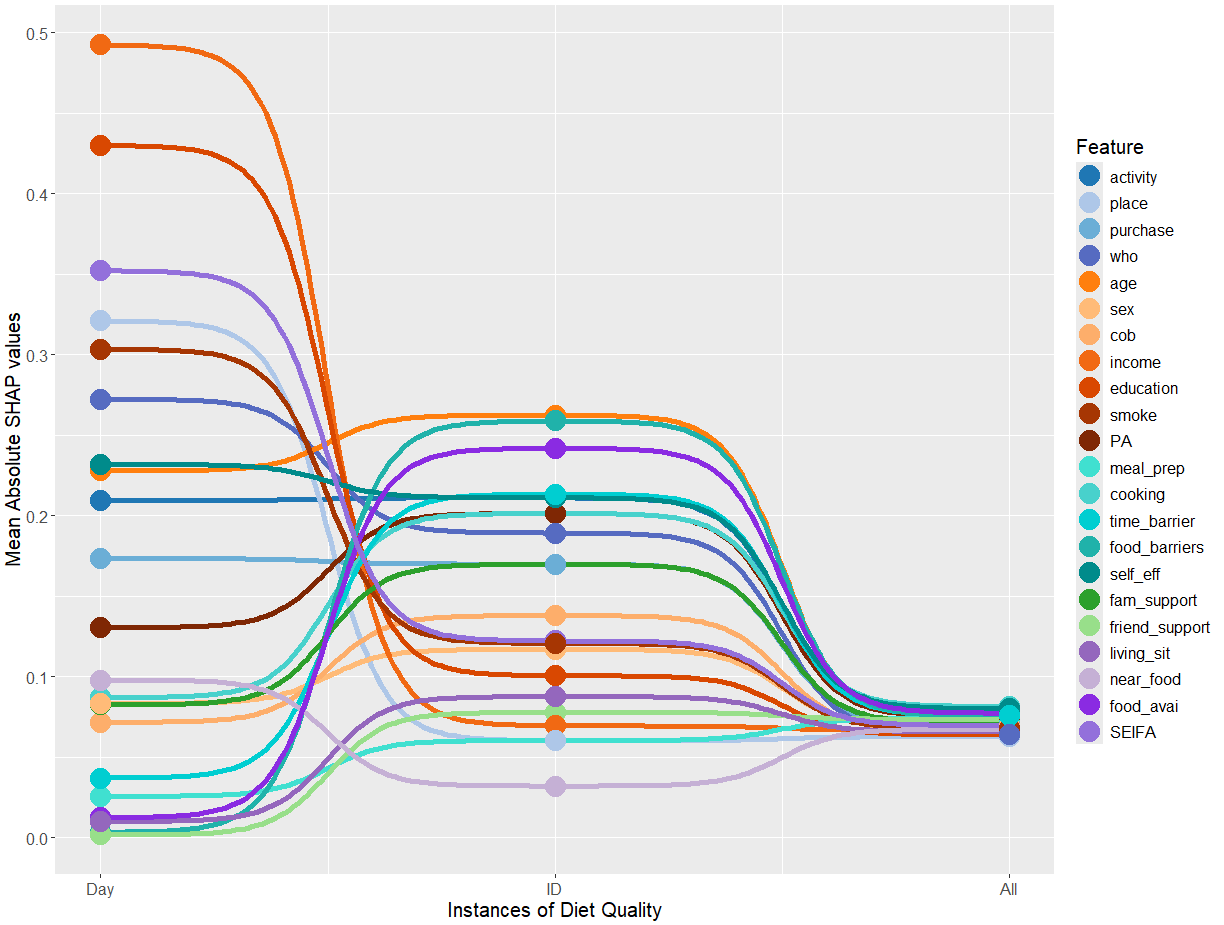


### Table A4.7 Mean Absolute SHAP values to assess predictors impact for predicting daily diet quality

| **Contextual factors** | **Eating Occasion** | **Day** | **Individual** | **All** |
| --- | --- | --- | --- | --- |
| Person-level: Intrapersonal factors |  |  |  |  |
| Age | 0.2278 | 0.2624 | 0.0741 | 0.2278 |
| Sex | 0.0833 | 0.1169 | 0.0689 | 0.0833 |
| Country of birth | 0.0716 | 0.1379 | 0.0668 | 0.0716 |
| Income | 0.4924 | 0.0693 | 0.0665 | 0.4924 |
| Education | 0.4299 | 0.1005 | 0.0638 | 0.4299 |
| Smoking status | 0.3033 | 0.1206 | 0.0678 | 0.3033 |
| Self-efficacy | 0.2317 | 0.2111 | 0.0800 | 0.2317 |
| Meal Preparation behaviour (food involvement) | 0.0256 | 0.0602 | 0.0741 | 0.0256 |
| Cooking confidence | 0.0869 | 0.2013 | 0.0814 | 0.0869 |
| Perceived time scarcity | 0.0369 | 0.2133 | 0.0760 | 0.0369 |
| Food choice barriers | 0.0032 | 0.2588 | 0.0752 | 0.0032 |
| Physical activity | 0.1304 | 0.2013 | 0.0752 | 0.1304 |
| Person-level: Socio-environmental factors |  |  |  |  |
| Social support from family | 0.0824 | 0.1697 | 0.0714 | 0.0824 |
| Social support from friends/colleagues | 0.0016 | 0.0777 | 0.0735 | 0.0016 |
| Person-level: Physical environmental factors |  |  |  |  |
| Proximity and access to food destinations | 0.0979 | 0.0320 | 0.0673 | 0.0979 |
| Food availability | 0.0122 | 0.2418 | 0.0772 | 0.0122 |
| Living situation | 0.0100 | 0.0875 | 0.0658 | 0.0100 |
| Area-level socio-economic position (SEIFA) | 0.3521 | 0.1221 | 0.0696 | 0.3521 |
| Eating occasion-level: Environmental factors |  |  |  |  |
| Place of consumption | 0.3209 | 0.0601 | 0.0626 | 0.3209 |
| Location of purchase | 0.1732 | 0.1699 | 0.0694 | 0.1732 |
| Eating occasion-level: Social factors |  |  |  |  |
| Presence of others | 0.2722 | 0.1891 | 0.0642 | 0.2722 |
| Activity at consumption | 0.2094 | 0.2110 | 0.0753 | 0.2094 |
